# Synthesis, crystal structure and Hirshfeld analysis of the bis­{(*E*)-2-[1-(benzo[*d*][1,3]dioxol-5-yl)ethylidene]-*N*-ethyl­hydrazine-1-carbo­thio­amide-κ*S*}di­chlorido­mercury(II) complex

**DOI:** 10.1107/S2056989026004822

**Published:** 2026-05-15

**Authors:** Renan Lira de Farias, Johannes Beck, Jörg Daniels, Adriano Bof de Oliveira

**Affiliations:** a Departamento de Química, Pontifícia Universidade Católica do Rio de Janeiro, Rua Marquês de São Vicente 225, 22451-900 Rio de Janeiro-RJ, Brazil; b Institut für Anorganische Chemie, Rheinische Friedrich-Wilhelms-Universität Bonn, Gerhard-Domagk-Strasse 1, D-53121 Bonn, Germany; c Núcleo de Química, Universidade Federal do Rio Grande, Avenida Itália km 08, 96203-900 Rio Grande-RS, Brazil; Universität Greifswald, Germany

**Keywords:** crystal structure, thio­semicarbazone complex, mercury(II) complex, H-bonded chelate-type, H-bonded intra­molecular rings

## Abstract

The title compound, [HgCl_2_(C_12_H_15_N_3_O_2_S)_2_], was synthesized by the reaction of (*E*)-2-[1-(benzo[*d*][1,3]dioxol-5-yl)ethyl­idene]-*N*-ethyl­hydrazine-1-carbo­thio­amide [common name: 3′,4′-(methyl­enedi­oxy)aceto­phenone 4-ethyl­thio­semicarbazone] with mercury(II) chloride in a 2:1 molar ratio at ambient temperature, using ethanol as solvent. A white solid was isolated and colourless single crystals of the title compound for the SC-XRD analysis were obtained from a solution in (methane­sulfin­yl)methane (common name: dimethyl sulfoxide, DMSO) with a hexane overlay. The Hg^II^ metal center is fourfold coordinated in a distorted tetra­hedral geometry by two neutral thio­semicarbazone derivatives, acting as κ*S*-donors, and two chlorido ligands. The complex mol­ecule exhibits a sequence of four intra­molecular hydrogen-bonded rings, formed by two inter­actions of the N—H⋯Cl type, with graph-set motifs of *S*(6) and showing a chelate-like coordination environment around the metal center, and two additional inter­actions of the N—H⋯N type with *S*(5) graph-set motifs. In the crystal, the mol­ecules are linked *via* N—H⋯Cl inter­molecular inter­actions along the *c*-axis, forming a hydrogen-bonded ribbon-like supra­molecular arrangement that resembles a zigzag pattern along the *c*-axis direction, and with the Cl atoms acting as bridges between intra- and inter­molecular hydrogen bonds. The Hirshfeld surface analysis suggests that the major contributions for the crystal cohesion are the H⋯H (40.4%), H⋯Cl/Cl⋯H (14.5%), H⋯C/C⋯H (11.3%), H⋯O/O⋯H (10.6%) and H⋯S/S⋯H (9.2%) inter­molecular contacts, with the surface being mapped over the *d*_norm_, shape-index and curvedness properties.

## Abstract 2

The synthesis, crystal structure and Hirshfeld analysis of the Hg^II^ complex with two mol­ecules of (*E*)-2-[1-(benzo[*d*][1,3]dioxol-5-yl)ethyl­idene]-*N-*ethyl­hydrazine-1-carbo­thio­amide, acting as κ*S*-donors, and two chlorido ligands are reported. For the first time, the structural N—H⋯Cl intra­molecular inter­actions for TSC–mercury(II) complexes are addressed as a chelate-like coordination environment with *S*(6) graph-set motifs.

## 1. Chemical context

Thio­semicarbazone derivatives, from now on TSCs, are organic mol­ecules with the functional group *R*_1_*R*_2_C=N—(H)N—C(=S)—N*R*_3_*R*_4_ and were first reported by Freund & Schander (1902 ▸) as products of a synthetic methodology for the organic qualitative analysis of aldehydes and ketones. For example, the thio­semicarbazide mol­ecule, H_2_N—N(H)—C(=S)—NH_2_, was employed as an analytical reagent for the detection of *R*_1_*R*_2_C=O and *R*_1_HC=O functional groups, with the respective TSC and H_2_O as products of the reaction. This classic condensation reaction with a nucleophilic attack from a Lewis base (H_2_N—*R*) to a carbonyl group (C=O) remains up to the present time a standard synthetic methodology for new compounds with a wide range of applications in several disciplines, including medicinal chemistry.
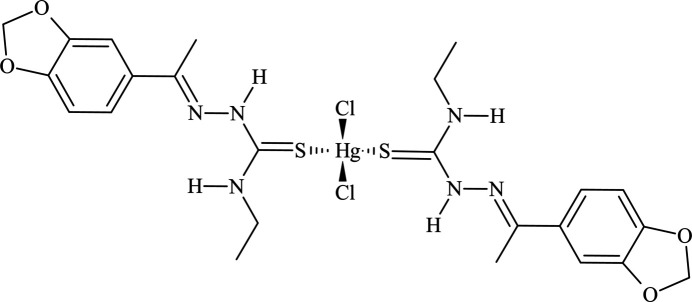


To the best of our knowledge, one of the first reports regarding the biological activity of TSCs, in this case for tuberculosis therapy, was published by Domagk *et al.* (1946 ▸). For other examples of the biological activities of TSCs and related compounds, see: Gupta *et al.* (2022 ▸); Khan *et al.* (2022 ▸); Pavan *et al.* (2010 ▸) and Parrilha *et al.* (2022 ▸).

Concerning the application of TSCs on coordination chemistry, some of the first reports are from Neuberg & Neimann (1902 ▸), describing the synthesis of TSC compounds with Ag^I^, and Kuhn & Zilliken (1954 ▸), showing the synthesis of TSC ligands with Cu^II^ for medicinal applications. For a review addressing complexes with TSC ligands, see: Lobana *et al.* (2009 ▸).

As part of our work on the TSCs coordination chemistry, we report herein the synthesis, crystal structure and Hirshfeld analysis of the title compound, from now on **HgCl_2_(TSC1)_2_**, including a discussion about the intra­molecular hydrogen bonds and their effects on the coordination sphere of TSC complexes with mercury(II) metal centers and chlorido ligands.

## 2. Structural commentary

**Figure 1:**
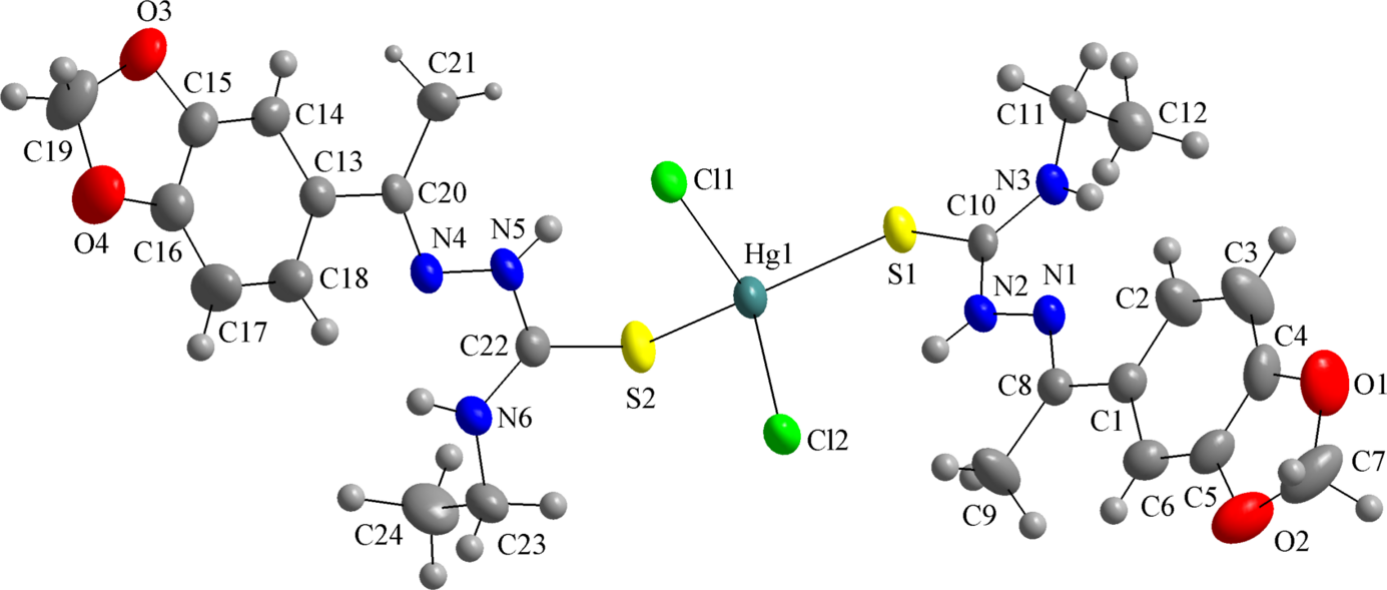
The mol­ecular structure of the title compound showing the atom labelling for all non-hydrogen atoms. The displacement ellipsoids are drawn at the 35% probability level and the H atoms are drawn in the ball-and-stick model for clarity.

**Figure 2:**
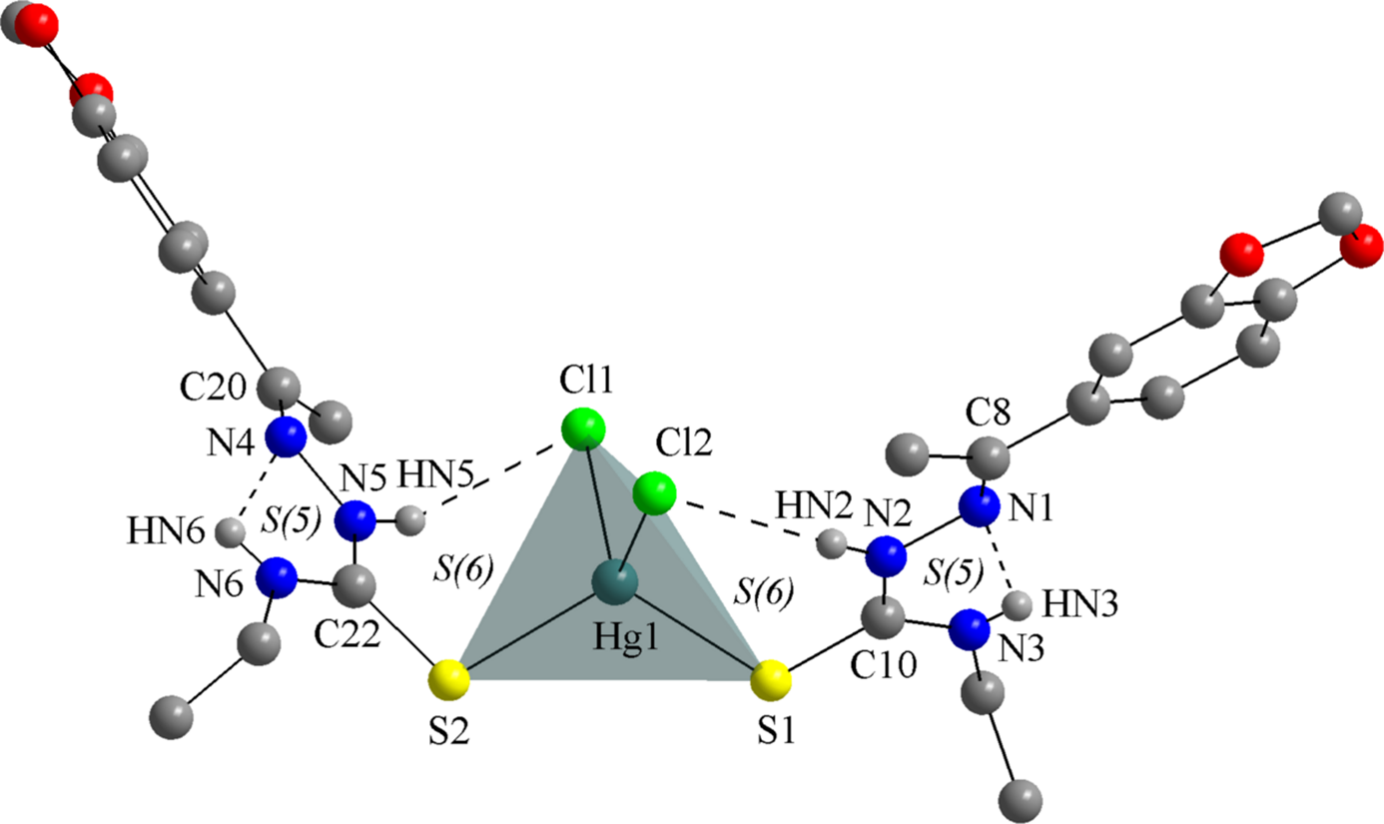
The mol­ecular structure of the title compound presented as ball-and-stick model, with the H⋯Cl and H⋯N intra­molecular inter­actions drawn as dashed lines and forming a sequence of four hydrogen-bonded rings with graph-set motifs of *S*(5) and *S*(6). The *S*(6) motifs resemble a hydrogen-based chelate-like coordination environment. The distorted tetra­hedral coordination polyhedron is drawn with 70% transparency and only key atoms are labelled. The figure is simplified for clarity.

The asymmetric unit of the title compound matches the mol­ecular formula, with all atoms located in general positions. The Hg^II^ metal center is fourfold coordinated in a distorted tetra­hedral geometry by two 3′,4′-(methyl­enedi­oxy)aceto­phenone 4-ethyl­thio­semicarbazones, **TSC1**, and two chlorido ligands (Fig. 1 ▸). The bond lengths and angles of the coordination sphere are in agreement with literature data for similar compounds and the respective values are given in Tables 1 ▸ and 2 ▸. For the Hg^II^ environment, a chelate-type coordination mode can be suggested based on the intra­molecular hydrogen-bonds, N2—H2*N*⋯Cl2 and N5—H5*N*⋯Cl1, with graph-set motifs of *S*(6), resulting in two six-membered H-based metallarings. As a result of these structural features, the spatial orientation of the **TSC1** ligands in the title compound can be assumed as a *V*-shape (Fig. 2 ▸). The formation of intra­molecular hydrogen bonds contributes to the thermodynamic stability of the mol­ecules (Koll *et al.*, 2006 ▸; Steiner, 2002 ▸) and it can be suggested that they compensate possible steric hindrance effects and lower mol­ecular symmetry, being a key structural feature for the complex addressed in this work. Additionally, two potential, albeit at rather acute angles, hydrogen-bonding contacts of the N—H⋯N type, *viz*, N3—H*N*3⋯N1 and N6—H*N*6⋯N4, with graph-set motifs *S*(5), are observed, forming a sequence of four hydrogen-bonded rings connected through the N2—C10 and N5—C22 bonds (Fig. 2 ▸ and Table 3 ▸).

One thio­semicarbazone fragment is almost planar, with the maximum deviation of the mean plane through the C8/N1/N2/C10/S1/N3 atoms being 0.0817 (39) Å for N1 (r.m.s.d. = 0.0449 Å). The entire ligand, however, is not planar due to the angle between the plane through the thio­semicarbazone moiety and the plane of the respective aromatic ring, which amounts to 16 (3)°, and the torsion angle regarding the terminal ethyl fragment, C10—N3—C11—C12, of −93.9 (8)°. The other thio­semicarbazone ligand is also not planar, with the C20/N4/N5/C22/S2 moiety showing the maximum deviation of the mean plane through the selected atoms of 0.1524 (39) Å for N4 (r.m.s.d. = 0.0774 Å), the angle between this plane and the respective aromatic ring of 31.4 (3)°, and the torsion angle regarding the respective ethyl group, C22—N6—C23—C24, being −106.9 (8)°.

**Figure 3:**
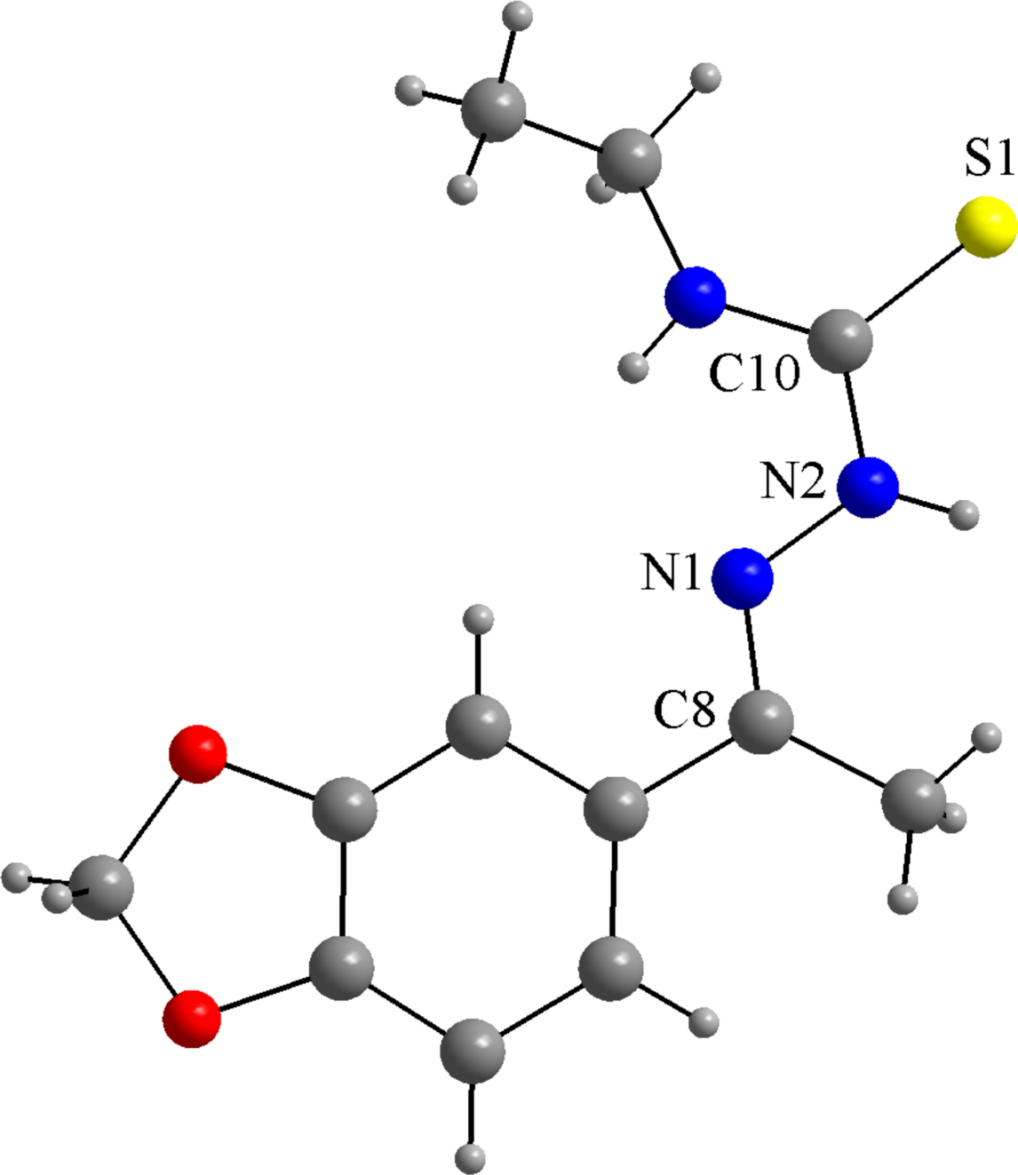
The mol­ecular structure of **TSC1** (CUCZUX; de Oliveira *et al.*, 2015 ▸) drawn as a ball-and-stick model and with only key atoms labelled. The disorder of the ethyl moiety is omitted for clarity.

The neutral form of **TSC1** is evident in the presence of the hydrazinic hydrogen atoms, H*N*2 and H*N*5, and the well-defined N—N, N—C and C=S bonds (Table 2 ▸; CSD refcode: CUCZUX; de Oliveira *et al.*, 2015 ▸; Fig. 3 ▸). In the deprotonated thio­semicarbazones, the hydrazinic H atom is removed and the resulting negative charge is delocalized over the N—C—S chain, in which the bond lengths tend to converge, with the N—N and N—C entities showing a double bond character and the C—S acquiring a single bond character (DAWTAZ; de Oliveira *et al.*, 2017 ▸ and TOKDUU; de Oliveira *et al.*, 2014 ▸). In the title compound, the C=S bond lengths are slightly longer than the respective bond in the crystal structure of the free ligand, **TSC1**, and this effect can be understood by the coordination of the thio­carbonyl groups to the metal center. The polarization of the electron density of the sulfur atoms toward the metal ion affects the C—S bond distances, as observed for similar compounds (Table 1 ▸). This effect is observed even for neutral TSC ligands, where the C—S bonds will not become single ones, but the related inter­atomic distances will increase by *ca*. 0.1 Å, which is a typical structural feature for these complexes.

## 3. Supra­molecular features

**Figure 4:**
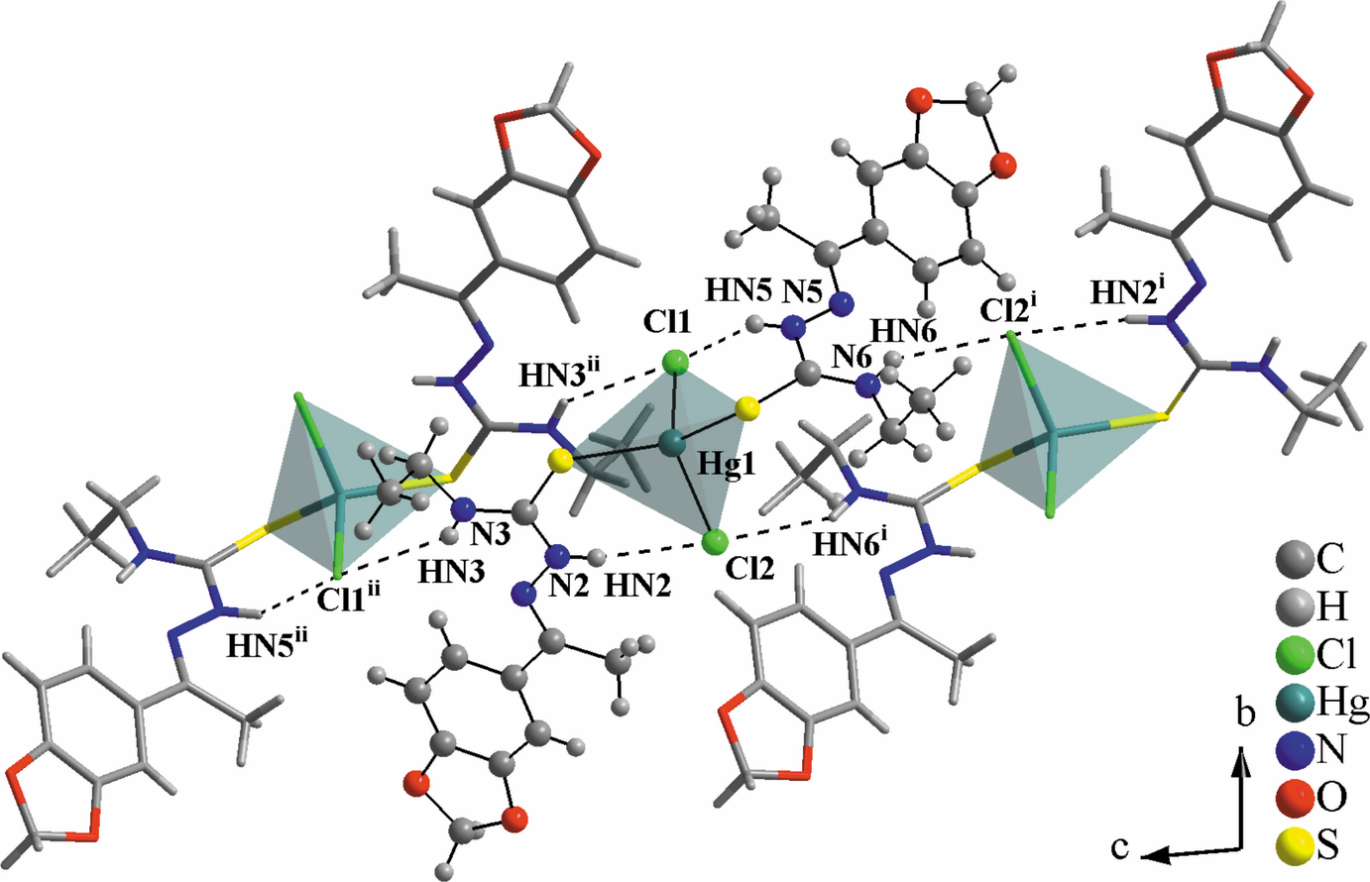
Section of the crystal structure of the title compound viewed along the *a* axis, showing the H⋯Cl inter­actions drawn as dashed lines and the mol­ecules linked into an 1-D ribbon-like supra­molecular arrangement along the *c*-axis direction. The asymmetric unit is presented as a ball-and-stick model, the distorted tetra­hedral coordination polyhedra are drawn with 70% transparency, and the figure is simplified for clarity. [Symmetry codes: (i) −*x* + 1, −*y* + 1, −*z* − 1; (ii) −*x* + 1, −*y* + 1, −*z*.]

**Figure 5:**
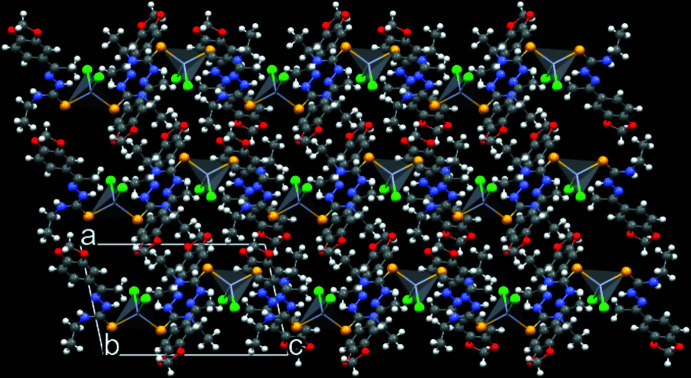
Graphical representation of the 3 × 3 × 3 expanded unit cell of the title compound viewed along the *b* axis, showing a zigzag pattern for the supra­molecular arrangement along the *c*-axis direction. For clarity, the mol­ecules are drawn using the ball-and-stick model and the coordination polyhedra are shown with 80% transparency.

In the crystal, the mol­ecules of the title compound are connected *via* N—H⋯Cl inter­molecular inter­actions, building a one-dimensional ribbon-like supra­molecular arrangement along the *c*-axis direction in which the Cl atoms act as bridges for intra- and inter­molecular hydrogen bonds, namely, H*N*3⋯Cl1^i^⋯H*N*5^i^ and H*N*6⋯Cl2^ii^⋯H*N*2^ii^ [symmetry codes: (i) −*x* + 1, −*y* + 1, −*z* − 1; (ii) −*x* + 1, −*y* + 1, −*z*] and the inter­action angles amount to 157.087 (3) and 170.750 (3)°, respectively (Fig. 4 ▸, Table 3 ▸). The H⋯Cl inter­atomic distances range from 2.55 to 2.79 Å, being shorter than the sum of the van der Waals radii for the respective atoms (2.95 Å according to Bondi, 1964 ▸; 2.86 Å by Rowland & Taylor, 1996 ▸; from 2.86 Å to 3.06 Å, as compiled by Batsanov, 2001 ▸). In addition, concerning the supra­molecular arrangement of the title compound, a zigzag pattern along the *c*-axis direction is observed in a 3 × 3 × 3 expanded unit cell, when viewed along the *b* axis (Fig. 5 ▸).

**Figure 6:**
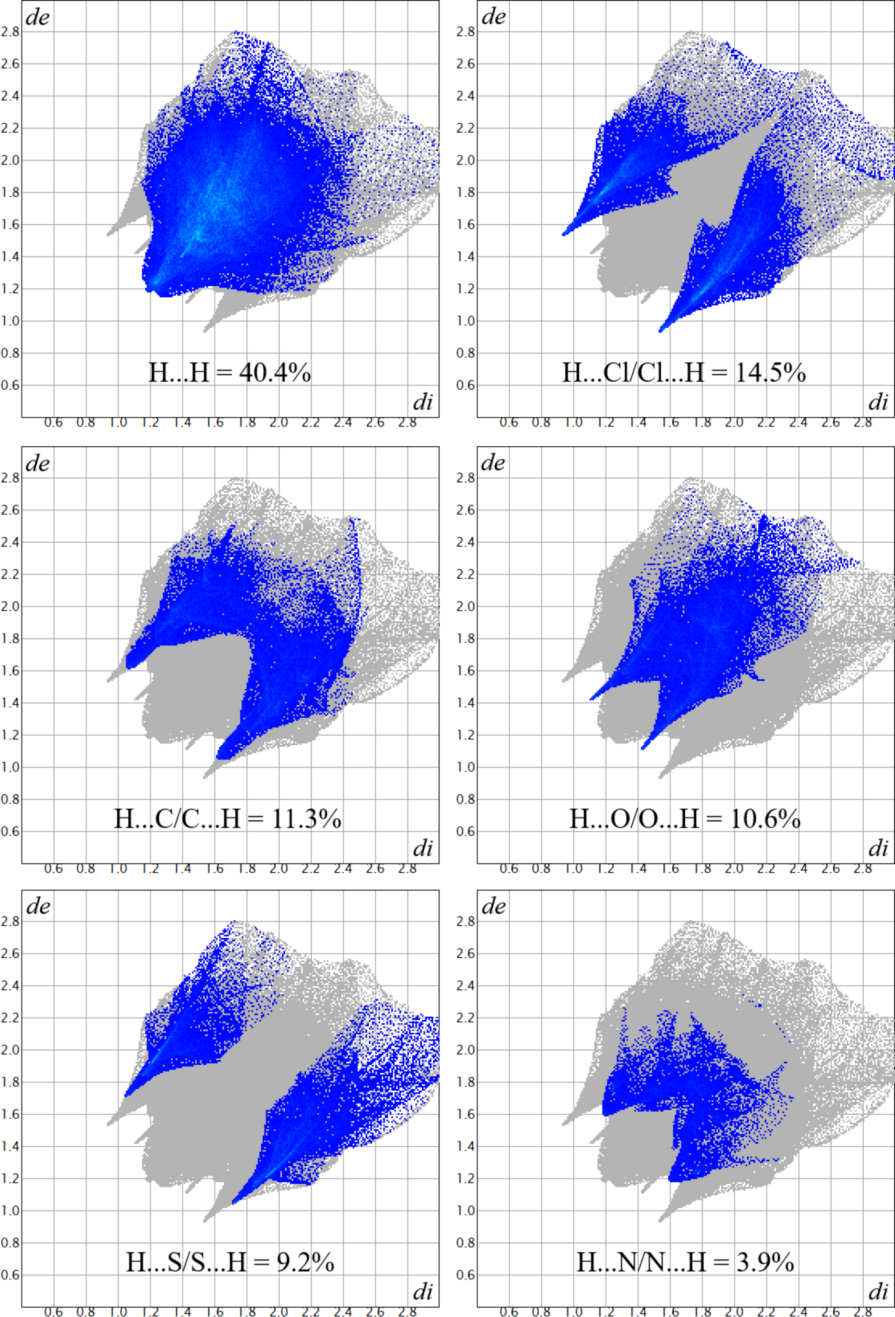
The HSFP-graphical representation of selected inter­molecular inter­action contributions for the crystal cohesion of the title compound. Inter­actions are highlighted in blue tones and contribution values (in %) are given within the figure. The *d*_e_ and *d*_i_ components are given in Å.

**Figure 7:**
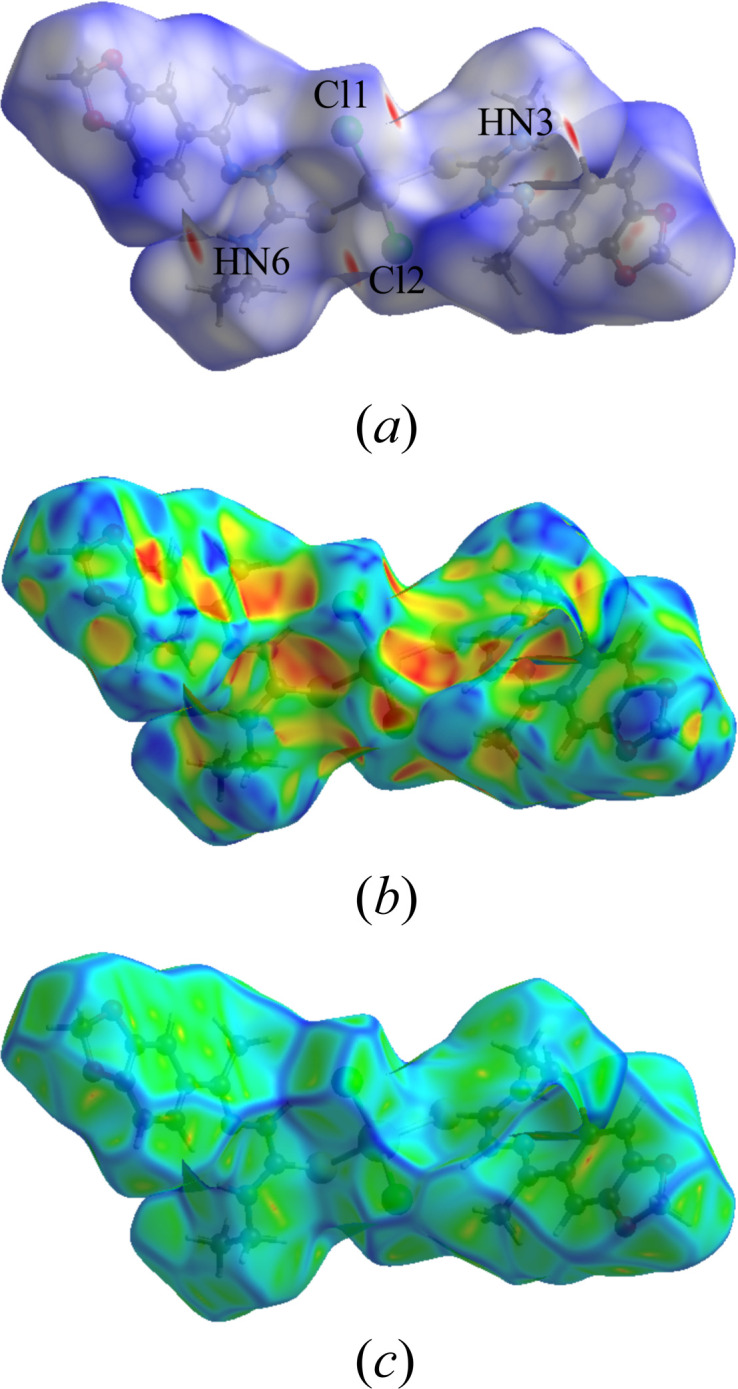
Graphical representations of the Hirshfeld surfaces of the title compound mapped over the following properties: (*a*) *d*_norm_ (range: 0,2655 to 1,4904 a.u.), with key atoms labelled and regions with strong inter­molecular contacts drawn in red, (*b*) shape-index (range: −1.0000 to 1.0000 a.u.), with the blue/concave regions for the donor atoms, the red/convex ones for acceptor atoms, and (*c*) curvedness (range: −4.0000 to 0.4000 a. u.), showing vertices and irregularities over the aromatic rings, suggesting that short-range inter­actions such as π–π contacts are absent.

An analysis of the inter­molecular inter­actions of the title compound was further performed with a Hirshfeld surface (Hirshfeld, 1977 ▸) evaluation, including the two-dimensional Hirshfeld surface fingerprints (HSFP) of the major contributions for the crystal cohesion and the graphical representations of the surface mapped over the *d*_norm_, shape-index and curvedness properties (Mackenzie *et al.*, 2017 ▸; Turner *et al.*, 2017 ▸). The most important contributions to the crystal packing are from H⋯H (40.4%), H⋯Cl/Cl⋯H (14.5%), H⋯C/C⋯H (11.3%), H⋯O/O⋯H (10.6%) and H⋯S/S⋯·H (9.2%) inter­molecular contacts. To complete the series with hydrogen atoms, the H⋯N/N⋯H (3.9%) contacts were included in the analysis and the results are presented in a single figure, with the contact types and contributions given within the graphics (Fig. 6 ▸). The Hirshfeld surface mapped over *d*_norm_ shows in red the regions related to strong inter­molecular contacts, corresponding in this work to the regions around the H3*N*, H6*N*, Cl1 and Cl2 atoms, as shown in Fig. 7 ▸(*a*). The surface regions drawn in blue and white indicate locations with weak or irrelevant inter­molecular inter­actions. The surface mapping set to the shape-index mode indicates the regions of the donor atoms in blue and concave local surfaces and the regions of the acceptor ones in red and convex surfaces. In the crystal structure of the title compound, the regions around the H atoms are related to the donor atoms and the regions around the Cl atoms mainly as acceptors, as depicted in Fig. 7 ▸(*b*). Finally, the surface mapped over curvedness indicates regions proper for inter­molecular inter­actions as flat local surfaces, while regions in which close contacts are precluded are shown as surfaces with irregularities or vertices. For example, the local surfaces over the aromatic rings are not flat, which suggests that inter­molecular inter­actions such as π–π-stacking are strongly unlikely, as observed in Fig. 7 ▸(*c*).

## 4. Database survey

The database survey for the title compound was performed with the Cambridge Structural Database (CSD, accessed *via* the WebCSD tool on April 10, 2026; Groom *et al.*, 2016 ▸) and the *ConQuest* software (Version 2025.2.0, accessed on April 10, 2026; Bruno *et al.*, 2002 ▸), being refined with the following parameters: two neutral thio­semicarbazone fragments acting as κ*S*-donors and two chlorido ligands coordinated to an Hg^II^ metal center. The survey returned only four crystal structures, *viz.* the complex with the *p*-di­methyl­amino­benzaldehyde thio­semicarbazone derivative, **HgCl_2_(TSC2)_2_**, (EFUKEX; Trzesowska-Kruszynska, 2014 ▸), the complex with 2-thio­phene­aldehyde-*N*(4)-naphthyl­thio­semicarbazone, **HgCl_2_(TSC3)_2_**, (IRETOP; Basu & Das, 2011 ▸), the complex with 2-(anthracen-9-yl­methyl­ene)-*N-*phenyl­thio­semicarbazone,**HgCl_2_(TSC4)_2_**, (MOCXAH; Nath & Baruah, 2023 ▸) and the complex with benzaldehyde-*N*(4),*N*(4)-di­methyl­thio­semi­carbazone, **Hg_2_Cl_4_(TSC5)_2_**, (GUTLEN; López-Torres & Mendiola, 2010 ▸).

**Figure 8:**
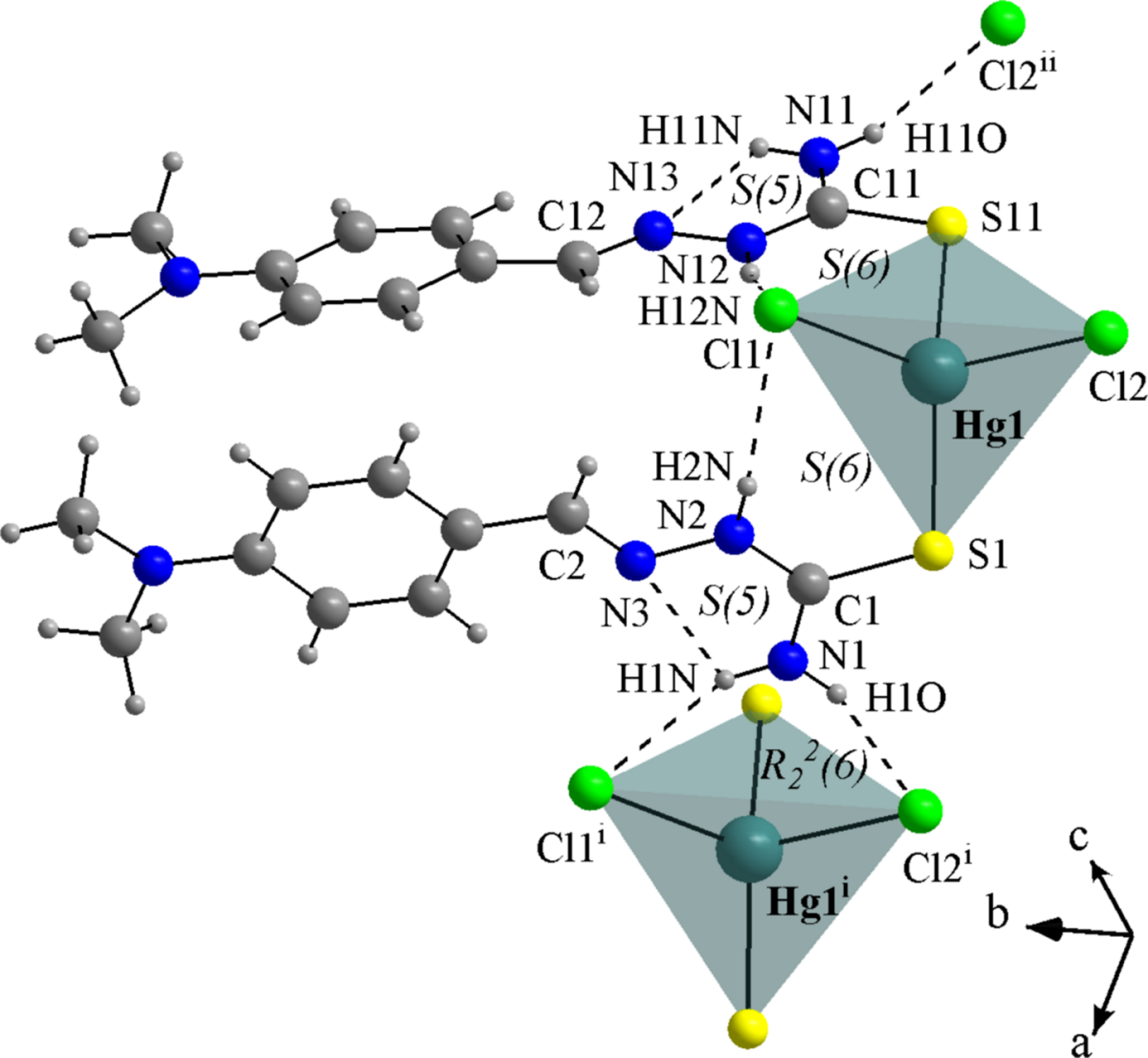
Section of the crystal structure of **HgCl_2_(TSC2)_2_** (EFUKEX; Trzesowska-Kruszynska, 2014 ▸), with the H-bonding being drawn as dashed lines. The structure is expanded by the N1—H1*N*⋯Cl^i^ and N1—H1*O*⋯Cl2^i^ inter­actions, forming a graph-set motif of 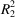
(6) and a chelate-type environment for the Hg1^i^ center, and by the N11—H11*O*⋯Cl2^ii^ inter­action into a hydrogen-bonded tape-like supra­molecular arrangement along the *ac*-plane. A ball-and-stick model is used for the graphical representation, which is simplified for clarity. Only key atoms are labelled and the distorted tetra­hedral coordination polyhedra are drawn with 70% transparency. [Symmetry codes: (i) *x* + 1, *y*, *z*; (ii) *x*, −*y* + 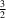, *z* + 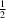.]

**Figure 9:**
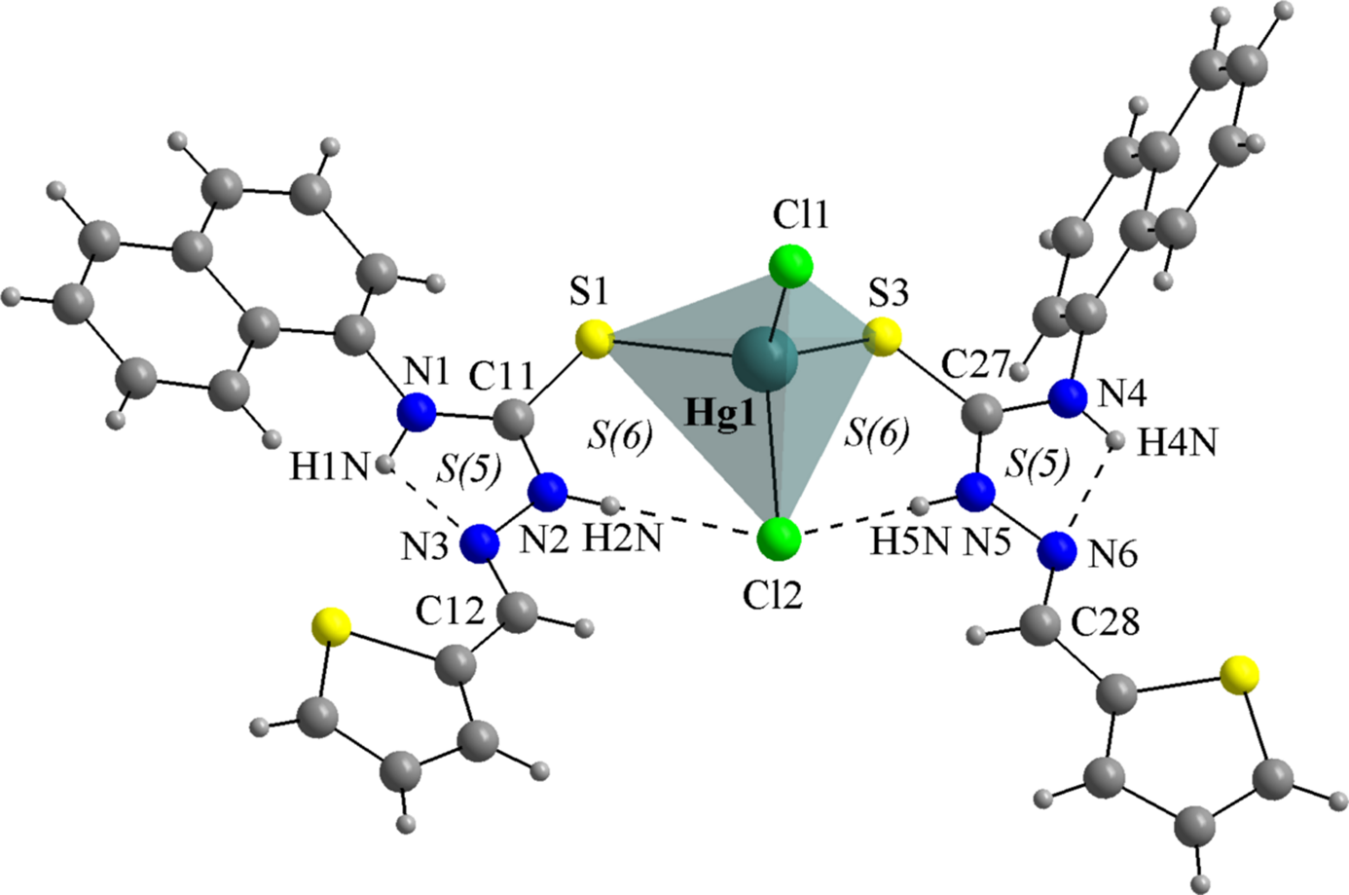
The mol­ecular structure of **HgCl_2_(TSC3)_2_** (IRETOP; Basu & Das, 2011 ▸). The intra­molecular hydrogen bonds are drawn as dashed lines. A ball-and-stick model is used for the graphical representation; only key atoms are labelled and the distorted tetra­hedral coordination polyhedron is drawn with 70% transparency for clarity.

**Figure 10:**
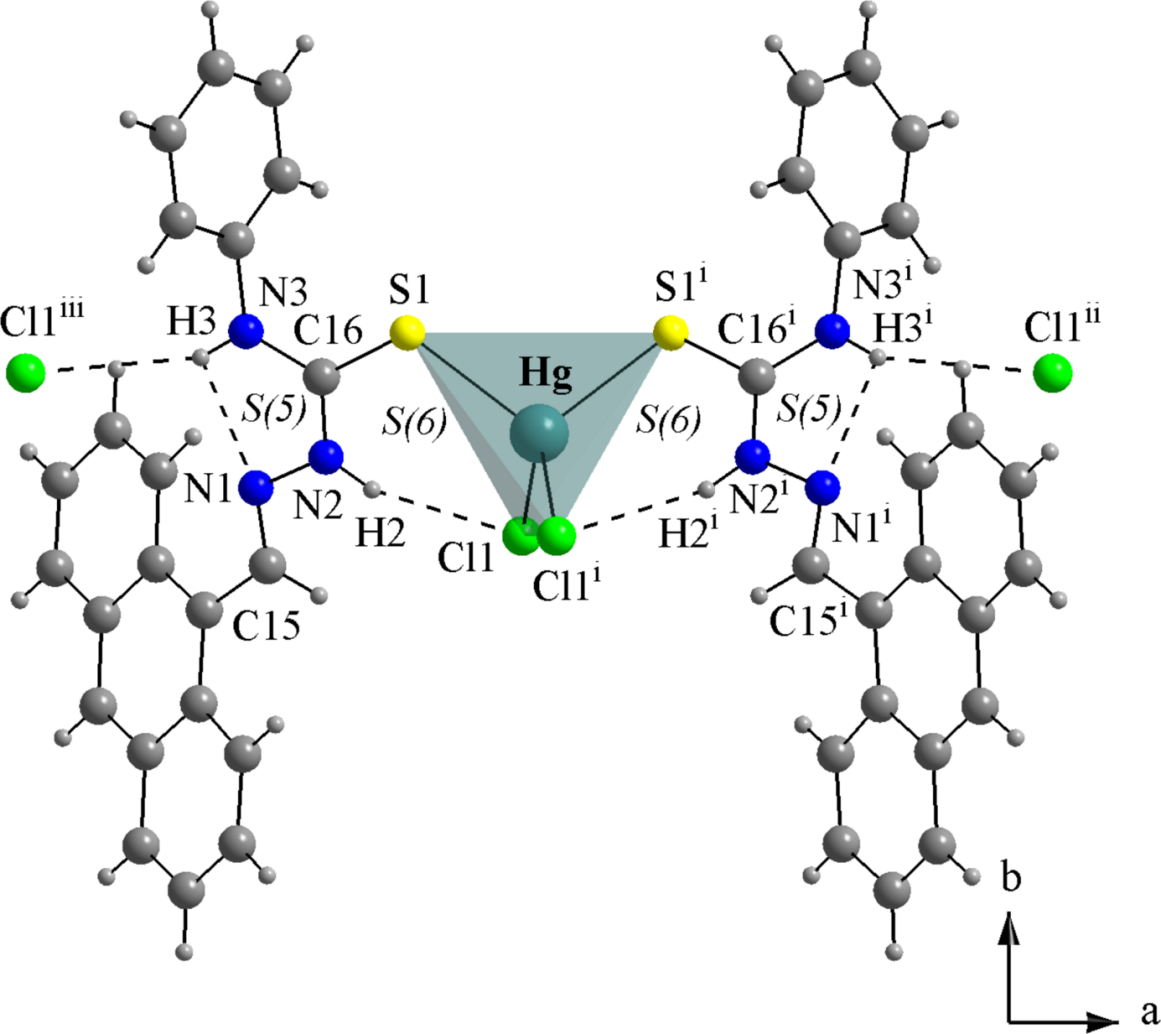
Crystal structure section of **HgCl_2_(TSC4)_2_** (MOCXAH; Nath & Baruah, 2023 ▸), in which the H-bonding is drawn as dashed lines. The mol­ecules are linked by N—H⋯Cl inter­actions, building a 1-D ribbon-like supra­molecular arrangement along the *a*-axis direction. A ball-and-stick model is used for the graphical representation; only key atoms are labelled and the distorted tetra­hedral coordination polyhedron is drawn with 70% transparency for clarity. [Symmetry codes: (i) −*x* + 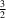, *y*, −*z* + 1; (ii) −*x* + 2, −*y* + 1, −*z* + 1; (iii) *x* − 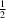, −*y* + 1, *z*.]

**Figure 11:**
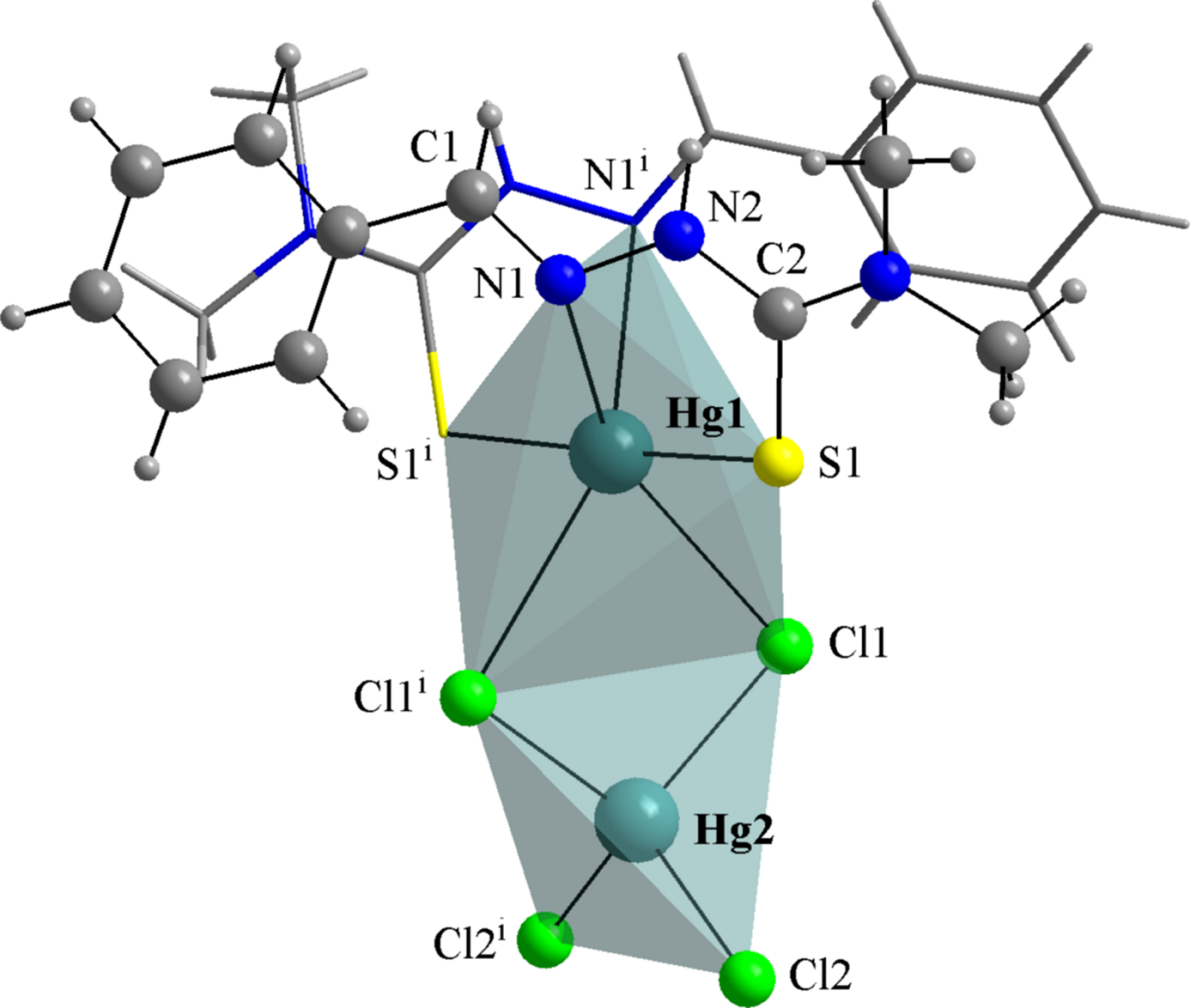
The mol­ecular structure of **Hg_2_Cl_4_(TSC5)_2_** (GUTLEN; López-Torres & Mendiola, 2010 ▸). A ball-and-stick model is used for the graphical representation; only key atoms are labelled and the distorted coordination polyhedra are drawn with 70% transparency for clarity. [Symmetry code: (i) −*x*, *y*, −*z* + 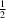.]

For the mol­ecular structures of **HgCl_2_(TSC2)_2_** (Fig. 8 ▸), **HgCl_2_(TSC3)_2_** (Fig. 9 ▸) and **HgCl_2_(TSC4)_2_** (Fig. 10 ▸), the Hg^II^ metal center is fourfold coordinated by two neutral thio­semicarbazones acting as κ*S*-donors and two chlorido ligands, exhibiting a similar coordination environment as observed for the title compound (Figs. 1 ▸ and 2 ▸). The distorted tetra­hedral geometries are assured by the respective Hg—Cl and Hg—S bond lengths (Table 1 ▸) and the selected bond angles (Table 2 ▸). For the title compound, all bond angles in the coordination sphere are reported, while for the **HgCl_2_(TSC2)_2_**, **HgCl_2_(TSC3)_2_** and **HgCl_2_(TSC4)_2_** complexes, only the maximal and the minimal values are given. The mol­ecular structure of **Hg_2_Cl_4_(TSC5)_2_** has a totally different coordination environment for the metal centers, being a dinuclear complex (Fig. 11 ▸), and the data given in Tables 1 ▸ and 2 ▸ refer to the asymmetric unit only.

The hydrogen-bonding geometries observed for the **HgCl_2_(TSC2)_2_**, **HgCl_2_(TSC3)_2_** and **HgCl_2_(TSC4)_2_** complexes (Figs. 8 ▸, 9 ▸ and 10 ▸; Table 4 ▸) adopt a pattern very similar to the title compound. This mol­ecular arrangement includes two intra­molecular inter­actions of the N—H⋯Cl type, with *S*(6) graph-set motifs forming a chelate-like coordination environment around the metal center. Two additional inter­actions of the N—H⋯N type, with *S*(5) graph-set motif may be considered. It must be pointed out, though, that for the three mol­ecules from the database the respective angles are even more acute than in the title compound, ranging from 103 to 108°. For **HgCl_2_(TSC2)_2_** and **HgCl_2_(TSC3)_2_**, the TSCs are oriented to the same side of the respective mol­ecules, possibly due to the H2*N*⋯Cl1⋯H12*N* and H2*N*⋯Cl2⋯H5*N* intra­molecular bridges. Regarding the supra­molecular arrangements, the **HgCl_2_(TSC2)_2_** mol­ecules are linked by N—H⋯Cl inter­molecular inter­actions into a tape-like structure along the *ac*-plane (Fig. 8 ▸, Table 4 ▸), while the **HgCl_2_(TSC4)_2_** mol­ecules are connected by the same type of inter­molecular inter­actions observed in this work, forming a one-dimensional ribbon-like structure along the *a*-axis direction (Fig. 10 ▸, Table 4 ▸). Neither strong nor relevant inter­molecular inter­actions were observed for **HgCl_2_(TSC3)_2_** and therefore only weak inter­molecular inter­actions, *e.g.*, the London dispersion forces can be suggested. Thus, the mol­ecules can be presented as discrete units in the crystal structure (for a graphical representation, see the supporting information). N.B. the *D*—H bond lengths in Table 4 ▸ were obtained directly from deposited data (984145, 793913 and 2233994 CIF files from CCDC *via* www.ccdc.cam.ac.uk/structures) while the other values were measured using *DIAMOND 3.2* (Brandenburg, 2006 ▸).

Lastly, in **Hg_2_Cl_4_(TSC5)_2_** one Hg^II^ metal center is coordinated by two neutral thio­semicarbazone derivatives in a chelate mode, acting as κ*N*,*S*-donors, and by two chlorido ligands, forming a six-vertex polyhedron resembling a strongly distorted octa­hedron. The second Hg^II^ center is coordinated by four chlorido ligands in a distorted tetra­hedral geometry (Fig. 11 ▸). The mercury(II) centers are connected by Cl atoms acting as bridges, *viz.* Hg1—Cl1—Hg2 and Hg1—Cl1^i^—Hg2, with the bond angle being 90.29 (4)° [symmetry code: (i) −*x*, *y*, −*z* + 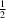]. Selected bond lengths and angles for comparison with the title compound and a neutral thio­semicarbazone derivative are given in Tables 1 ▸ and 2 ▸. Even though the mol­ecular structure of **Hg_2_Cl_4_(TSC5)_2_** does not exhibit intra­molecular hydrogen bonding and cannot contribute to the discussion about their effects on the coordination and mol­ecular geometries of TSC complexes addressed in this work, the report from López-Torres & Mendiola (2010 ▸) is still a notable reference for Hg_*x*_Cl_*y*_(TSC)_2_ compounds.

## 5. Synthesis and crystallization

The starting materials are commercially available and were used without further purification. The **TSC1** ligand was obtained as previously reported (de Oliveira *et al.*, 2015 ▸) and suspended in ethanol (1 mmol, 0.2653 g in 50 mL) under magnetic stirring at room temperature. Under the same conditions, a suspension of mercury(II) chloride in ethanol was prepared (0.5 mmol, 0.1357 g in 50 mL). The solutions were combined and stirred at room temperature for 4 h, after which a white solid was formed, and afterwards isolated by filtration. The one-step synthesis was adapted from literature procedures (Basu & Das, 2011 ▸; López-Torres & Mendiola, 2010 ▸; Nath & Baruah, 2023 ▸). The solid was washed with small portions of cold ethanol and dried at room temperature. As a result of the non-uniformity of the bulk white solid, the purification of the product and the yield determination were not possible. Colourless single crystals suitable for X-ray diffraction were obtained in a test tube from a solution of the solid in dimethyl sulfoxide with a hexane overlay after some weeks.

## 6. Refinement

Crystal data, data collection and structure refinement details are summarized in Table 5 ▸. The structure solution was performed using direct methods and refined on *F*^2^ with anisotropic displacement factors for the non-hydrogen atoms. The H-atoms were treated by a mixture of constrained and independent refinement. The H atoms attached to C7, C9, C19 and C21 (Fig. 1 ▸) were positioned with idealized geometry and refined applying the HFIX instruction, with *U*_iso_(H) = 1.2 *U*_eq_ (C7/C19 atoms) and *U*_iso_(H) = 1.5 *U*_eq_ (C9/C21 atoms), with bond lengths set to C—H = 0.97 and 0.96 Å, respectively. The remaining hydrogen atoms were located in difference-Fourier maps and freely refined with isotropic displacement parameters. The C—H bond lengths in the aromatic rings range from 0.87 (6) Å for C6—H6 to 1.05 (6) Å for C17—H17, while the values for the ethyl entities range from 0.90 (7) Å for C23—H23*B* to 1.21 (7) Å for C24—H24*A*. The N—H bond lengths range from 0.76 (5) Å, N3—H*N*3, to 0.87 (6) Å, N6—H*N*6. Planes through selected atoms, torsion angles and the hydrogen-bond geometries were calculated with the MPLA, CONF and HTAB instructions. Only classical hydrogen bonds were considered for the discussion in this work, while the complete dataset from the refinement is provided in Table 3 ▸.

## Supplementary Material

Crystal structure: contains datablock(s) I. DOI: 10.1107/S2056989026004822/yz2078sup1.cif

Structure factors: contains datablock(s) I. DOI: 10.1107/S2056989026004822/yz2078Isup2.hkl

Additional figures for the supramolecular arrangement of the title compound and the complexes of the literature survey. DOI: 10.1107/S2056989026004822/yz2078sup3.pdf

CCDC reference: 2552717

Additional supporting information:  crystallographic information; 3D view; checkCIF report

## Figures and Tables

**Table 1:** Selected bond lengths (Å) of the title compound and from literature data

Compounds	CSD refcodes	Chemical bonds	Bond lengths	Chemical bonds	Bond lengths
HgCl_2_(TSC1)_2_^*a*^	This work	N1—N2	1.390 (6)	N4—N5	1.399 (6)
		N2—C10	1.349 (7)	N5—C22	1.353 (7)
		C10—S1	1.725 (5)	C22—S2	1.720 (6)
		Hg1—Cl1	2.5681 (15)	Hg1—Cl2	2.5132 (15)
		Hg1—S1	2.4832 (14)	Hg1—S2	2.4878 (16)
					
TSC1^*b*^	CUCZUX	N1—N2	1.3730 (18)		
		N2—C10	1.358 (2)		
		C10—S1	1.6792 (17)		
					
HgCl_2_(TSC2)_2_^*c*^	EFUKEX	N3—N2	1.383 (4)	N13—N12	1.393 (4)
		N2—C1	1.330 (4)	N12—C11	1.307 (5)
		C1—S1	1.740 (3)	C11—S11	1.732 (4)
		Hg1—Cl1	2.7490 (8)	Hg1—Cl2	2.5947 (9)
		Hg1—S1	2.4049 (10)	Hg1—S11	2.4192 (9)
					
HgCl_2_(TSC3)_2_^*d*^	IRETOP	N2—N3	1.367 (10)	N5—N6	1.375 (9)
		N2—C11	1.342 (12)	N5—C27	1.321 (10)
		C11—S1	1.710 (9)	C27–S3	1.721 (9)
		Hg1—Cl1	2.397 (4)	Hg1—Cl2	2.607 (3)
		Hg1—S1	2.533 (3)	Hg1—S3	2.496 (2)
					
HgCl_2_(TSC4)_2_^*e*^	MOCXAH	N1—N2	1.392 (6)		
		N2—C16	1.328 (6)		
		C16—S1	1.715 (4)		
		Hg—Cl1	2.5177 (12)	Hg—S1	2.4975 (12)
					
Hg_2_Cl_4_(TSC5)_2_^*f*^	GUTLEN	N1—N2	1.379 (4)		
		N2—C2	1.343 (4)		
		C2—S1	1.727 (3)		
		Hg1—Cl1	3.0387 (12)	Hg1—S1	2.3732 (8)
		Hg1—N1	2.748 (3)		
		Hg2—Cl1	2.4888 (12)	Hg2—Cl2	2.4653 (10)

Notes: (*a*) this work [TSC1 is 3′,4′-(methyl­enedi­oxy)aceto­phenone 4-ethyl­thio­semicarbazone]; (*b*) de Oliveira *et al.* (2015 ▸); (*c*) Trzesowska-Kruszynska (2014 ▸) (TSC2 is *p*-di­methyl­amino­benzaldehyde thio­semicarbazone); (*d*) Basu & Das (2011 ▸) [TSC3 is 2-thio­phene­aldehyde-*N*(4)-naphthyl­thio­semicarbazone]; (*e*) Nath & Baruah (2023 ▸) [TSC4 is 2-(anthracen-9-yl­methyl­ene)-*N*-phenyl­thio­semicarbazone]; (*f*) López-Torres & Mendiola (2010 ▸) [TSC5 is benzaldehyde-*N*(4),*N*(4)-di­methyl­thio­semicarbazone].

**Table 2:** Selected bond angles (°) for the coordination sphere of Hg^II^ complexes with chlorido and thio­semicarbazone ligands

Compounds	CSD refcodes	Chemical bonds	Angles
HgCl_2_(TSC1)_2_^*a*^	This work	S1—Hg1—S2	117.71 (5)
		Cl2—Hg1—S1	109.19 (5)
		Cl1—Hg1—S1	109.03 (5)
		Cl1—Hg1—S2	108.61 (5)
		Cl2—Hg1—S2	107.93 (6)
		Cl1—Hg1—Cl2	103.42 (6)
			
HgCl_2_(TSC2)_2_^*b*^	EFUKEX	S1—Hg1—S11	150.70 (4)
		Cl2—Hg1—S11	93.18 (4)
			
HgCl_2_(TSC3)_2_^*c*^	IRETOP	Cl1—Hg1—S3	119.21 (17)
		Cl1—Hg1—Cl2	99.30 (12)
			
HgCl_2_(TSC4)_2_^*d*^	MOCXAH	Cl^i^’—Hg—S1^i^	114.71 (4)
		S1—-Hg—S1^i^	105.43 (6)
			
Hg_2_Cl_4_(TSC5)_2_^*e*^	GUTLEN	Cl1—Hg1—N1	109.65 (7)
		N1—Hg1—S1	106.72 (6)
		Cl1—Hg1—N1	79.48 (3)
		Cl1—Hg2—Cl2	110.65 (4)
		Hg1—Cl1—Hg2	90.29 (4)

Notes: (*a*) this work [TSC1 is 3′,4′-(methyl­enedi­oxy)aceto­phenone 4-ethyl­thio­semicarbazone]; (*b*) Trzesowska-Kruszynska (2014 ▸) (TSC2 is *p*-di­methyl­amino­benzaldehyde thio­semicarbazone); (*c*) Basu & Das (2011 ▸) [TSC3 is 2-thio­phene­aldehyde-*N*(4)-naphthyl­thio­semicarbazone]; (*d*) Nath & Baruah (2023 ▸) [TSC4 is 2-(anthracen-9-yl­methyl­ene)-*N*-phenyl­thio­semicarbazone]; (*e*) López-Torres & Mendiola (2010 ▸) [TSC5 is benzaldehyde-*N*(4),*N*(4)-di­methyl­thio­semicarbazone]. Symmetry code: (i) −*x* + 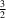, *y*, −*z* + 1.

**Table 3:** Hydrogen-bond geometry (Å, °)

*D*—H⋯*A*	*D*—H	H⋯*A*	*D*⋯*A*	*D*—H⋯*A*
C7—H7*B*⋯S1^i^	0.97	2.85	3.642 (8)	139
C9—H9*C*⋯Cl2	0.96	2.89	3.474 (7)	121
C21—H21*A*⋯Cl1	0.96	2.90	3.675 (6)	139
C21—H21*B*⋯Cl2^ii^	0.96	2.95	3.707 (6)	136
N2—H*N*2⋯Cl2	0.83 (5)	2.53 (5)	3.354 (5)	170 (4)
N3—H*N*3⋯Cl1^iii^	0.76 (5)	2.68 (5)	3.293 (5)	140 (4)
N3—H*N*3⋯N1	0.76 (5)	2.22 (5)	2.604 (6)	113 (4)
N5—H*N*5⋯Cl1	0.85 (7)	2.53 (7)	3.233 (6)	141 (6)
N6—H*N*6⋯Cl2^iv^	0.87 (6)	2.58 (6)	3.300 (5)	141 (5)
N6—H*N*6⋯N4	0.87 (6)	2.19 (6)	2.649 (7)	112 (5)
C23—H23*A*⋯S2	0.96 (6)	2.60 (6)	3.134 (8)	115 (4)
C24—H24*A*⋯Cl2^iv^	1.21 (7)	2.77 (7)	3.739 (9)	137 (4)
C24—H24*B*⋯O3^v^	1.01 (7)	2.60 (7)	3.513 (11)	151 (6)

Symmetry codes: (i) 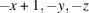; (ii) 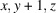; (iii) 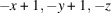; (iv) 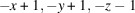; (v) 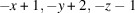.

**Table 4:** Hydrogen-bond geometry (Å, °) for the reference Hg^II^ complexes with chlorido and thio­semicarbazone ligands

Compounds	CSD refcodes	*D*—H⋯*A*	*D*—H	H⋯*A*	*D*⋯*A*	*D*—H⋯*A*
HgCl_2_(TSC2)_2_^*a*^	EFUKEX	N1—H1*N*⋯Cl1^i^	0.88	2.6242 (8)	3.4124 (3)	149.68 (20)
		N1—H1*O*⋯Cl2^i^	0.88	2.3794 (10)	3.2212 (32)	160.17 (20)
		N1—H1*N*⋯N3	0.88	2.2978 (29)	2.6384 (42)	102.98 (21)
		N11—H11*O*⋯Cl2^ii^	0.88	2.3555 (10)	3.2033 (38)	161.46 (23)
		N11—H11*N*⋯N13	0.88	2.2785 (30)	2.6241 (47)	103.26 (23)
		N12—H12*N*⋯Cl1	0.88	2.4057 (8)	3.2590 (29)	163.72 (18)
		N2—H2*N*⋯Cl1	0.88	2.3960 (8)	3.1684 (32)	146.66 (20)
						
HgCl_2_(TSC3)_2_^*b*^	IRETOP	N1—H1N⋯N3	0,88	2.1704 (83)	2.5671 (13)	107.82 (55)
		N2—H2*N*⋯Cl2	0.86	2.3558 (28)	3.2123 (96)	172.06 (62)
		N4—H4*N*⋯N6	0.88	2.2738 (83)	2.6350 (13)	105.31 (51)
		N5—H5*N*⋯Cl2	0.86	2.4030 (22)	3.2541 (66)	169.71 (41)
						
HgCl_2_(TSC4)_2_^*c*^	MOCXAH	N2—H2⋯Cl1	0.86	2.4803 (13)	3.2444 (47)	148.37 (30)
		N3—H3⋯Cl1^iii^	0.86	2.6802 (12)	3.3786 (32)	139.25 (20)
		N3—H3⋯N1	0.86	2.3039 (35)	2.6597 (46)	105.04 (22)

Notes: (*a*) Trzesowska-Kruszynska (2014 ▸) (TSC2 is *p*-di­methyl­amino­benzaldehyde thio­semicarbazone); (*b*) Basu & Das (2011 ▸) [TSC3 is 2-thio­phene­aldehyde-*N*(4)-naphthyl­thio­semicarbazone]; (*c*) Nath & Baruah (2023 ▸) [TSC4 is 2-(anthracen-9-yl­methyl­ene)-*N*-phenyl­thio­semicarbazone]. Symmetry codes: (i) *x* + 1, *y*, *z*; (ii) *x*, −*y* + 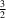, *z* + 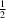; (iii) *x* − 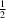, −*y* + 1, *z*.

**Table 5:** Experimental details

Crystal data	
Chemical formula	[HgCl_2_(C_12_H_15_N_3_O_2_S)_2_]
*M*_r_	802.15
Crystal system, space group	Triclinic, *P* 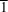
Temperature (K)	293
*a*, *b*, *c* (Å)	9.4412 (5), 10.4130 (5), 15.4626 (8)
α, β, γ (°)	94.616 (3), 101.797 (3), 91.079 (3)
*V* (Å^3^)	1482.24 (13)
*Z*	2
Radiation type	Mo *K*α
μ (mm^−1^)	5.55
Crystal size (mm)	0.08 × 0.08 × 0.07

Data collection	
Diffractometer	Enraf–Nonius FR590 Kappa CCD
Absorption correction	Analytical [using the algorithm from de Meulenaer & Tompa (1965 ▸), in Alcock (1970 ▸)]
*T*_min_, *T*_max_	0.634, 0.724
No. of measured, independent and observed [*I* > 2σ(*I*)] reflections	17004, 5627, 3849
*R*_int_	0.064
(sin θ/λ)_max_ (Å^−1^)	0.611

Refinement	
*R*[*F*^2^ > 2σ(*F*^2^)], *wR*(*F*^2^), *S*	0.036, 0.074, 0.97
No. of reflections	5627
No. of parameters	434
H-atom treatment	H atoms treated by a mixture of independent and constrained refinement
Δρ_max_, Δρ_min_ (e Å^−3^)	0.53, −1.29

Computer programs: *COLLECT* (Nonius, 1998 ▸), *HKL*, *DENZO* and *SCALEPACK* (Otwinowski & Minor, 1997 ▸), *SIR92* (Altomare *et al.*, 1994 ▸), *SHELXL2019/3* (Sheldrick, 2015 ▸), *CrystalExplorer 17.5* (Mackenzie *et al.*, 2017 ▸; Turner *et al.* 2017 ▸), *DIAMOND* (Brandenburg, 2006 ▸), *Mercury* (Macrae *et al.*, 2020 ▸), *WinGX* (Farrugia, 2012 ▸), *enCIFer* (Allen *et al.*, 2004 ▸) and *publCIF* (Westrip, 2010 ▸).

## supplementary crystallographic information

### Bis{(E)-2-[1-(benzo[d][1,3]dioxol-5-yl)ethylidene]-N-ethylhydrazine-1-carbothioamide-κS}dichloridomercury(II) Crystal data

**Table:**

[HgCl_2_(C_12_H_15_N_3_O_2_S)_2_]	*Z* = 2
*M**_r_* = 802.15	*F*(000) = 788
Triclinic, *P*1	*D*_x_ = 1.797 Mg m^−^^3^
*a* = 9.4412 (5) Å	Mo *K*α radiation, λ = 0.71073 Å
*b* = 10.4130 (5) Å	Cell parameters from 37747 reflections
*c* = 15.4626 (8) Å	θ = 2.9–25.7°
α = 94.616 (3)°	µ = 5.55 mm^−^^1^
β = 101.797 (3)°	*T* = 293 K
γ = 91.079 (3)°	Block, colourless
*V* = 1482.24 (13) Å^3^	0.08 × 0.08 × 0.07 mm

### Bis{(E)-2-[1-(benzo[d][1,3]dioxol-5-yl)ethylidene]-N-ethylhydrazine-1-carbothioamide-κS}dichloridomercury(II) Data collection

**Table:**

Enraf–Nonius FR590 Kappa CCD diffractometer	5627 independent reflections
Radiation source: sealed X-ray tube, Enraf–Nonius FR590	3849 reflections with *I* > 2σ(*I*)
Horizontally mounted graphite crystal monochromator	*R*_int_ = 0.064
Detector resolution: 9 pixels mm^-1^	θ_max_ = 25.7°, θ_min_ = 3.0°
CCD rotation images, thick slices, κ–goniostat scans	*h* = −9→11
Absorption correction: analytical [using the algorithm from de Meulenaer & Tompa (1965), in Alcock (1970)]	*k* = −12→12
*T*_min_ = 0.634, *T*_max_ = 0.724	*l* = −18→18
17004 measured reflections	

### Bis{(E)-2-[1-(benzo[d][1,3]dioxol-5-yl)ethylidene]-N-ethylhydrazine-1-carbothioamide-κS}dichloridomercury(II) Refinement

**Table:**

Refinement on *F*^2^	Primary atom site location: structure-invariant direct methods
Least-squares matrix: full	Secondary atom site location: difference Fourier map
*R*[*F*^2^ > 2σ(*F*^2^)] = 0.036	Hydrogen site location: mixed
*wR*(*F*^2^) = 0.074	H atoms treated by a mixture of independent and constrained refinement
*S* = 0.97	*w* = 1/[σ^2^(*F*_o_^2^) + (0.0254*P*)^2^] where *P* = (*F*_o_^2^ + 2*F*_c_^2^)/3
5627 reflections	(Δ/σ)_max_ < 0.001
434 parameters	Δρ_max_ = 0.53 e Å^−^^3^
0 restraints	Δρ_min_ = −1.29 e Å^−^^3^

### Bis{(E)-2-[1-(benzo[d][1,3]dioxol-5-yl)ethylidene]-N-ethylhydrazine-1-carbothioamide-κS}dichloridomercury(II) Special details

**Table:**

Geometry. All esds (except the esd in the dihedral angle between two l.s. planes) are estimated using the full covariance matrix. The cell esds are taken into account individually in the estimation of esds in distances, angles and torsion angles; correlations between esds in cell parameters are only used when they are defined by crystal symmetry. An approximate (isotropic) treatment of cell esds is used for estimating esds involving l.s. planes.

### Bis{(E)-2-[1-(benzo[d][1,3]dioxol-5-yl)ethylidene]-N-ethylhydrazine-1-carbothioamide-κS}dichloridomercury(II) Fractional atomic coordinates and isotropic or equivalent isotropic displacement parameters (Å^2^)

**Table:**

	*x*	*y*	*z*	*U*_iso_*/*U*_eq_	
C1	0.3201 (6)	0.0745 (5)	−0.0107 (4)	0.0493 (15)	
C2	0.3079 (9)	0.1267 (7)	0.0731 (5)	0.075 (2)	
C3	0.2253 (10)	0.0656 (9)	0.1249 (6)	0.095 (3)	
C4	0.1552 (7)	−0.0474 (7)	0.0891 (5)	0.069 (2)	
C5	0.1682 (6)	−0.0989 (5)	0.0070 (5)	0.0578 (17)	
C6	0.2461 (7)	−0.0429 (6)	−0.0440 (5)	0.0574 (18)	
C7	0.0225 (9)	−0.2283 (7)	0.0626 (7)	0.102 (3)	
H7A	−0.082097	−0.229416	0.043760	0.122*	
H7B	0.049474	−0.308882	0.087752	0.122*	
C8	0.4078 (6)	0.1421 (5)	−0.0636 (4)	0.0460 (14)	
C9	0.4074 (9)	0.0888 (7)	−0.1563 (4)	0.086 (2)	
H9A	0.325785	0.119674	−0.195928	0.130*	
H9B	0.401075	−0.003678	−0.159620	0.130*	
H9C	0.495177	0.116033	−0.172778	0.130*	
C10	0.6490 (5)	0.4092 (5)	−0.0252 (3)	0.0381 (13)	
C11	0.7496 (7)	0.5229 (6)	0.1225 (4)	0.0493 (15)	
C12	0.8945 (8)	0.4736 (8)	0.1636 (6)	0.0673 (19)	
C13	0.2798 (6)	0.9187 (5)	−0.5172 (4)	0.0486 (15)	
C14	0.2181 (7)	1.0385 (6)	−0.5100 (4)	0.0508 (16)	
C15	0.1042 (7)	1.0655 (6)	−0.5742 (4)	0.0562 (16)	
C16	0.0471 (7)	0.9789 (7)	−0.6435 (5)	0.0688 (19)	
C17	0.1021 (9)	0.8602 (8)	−0.6533 (5)	0.087 (3)	
C18	0.2212 (8)	0.8313 (7)	−0.5881 (5)	0.071 (2)	
C19	−0.0681 (8)	1.1626 (7)	−0.6631 (6)	0.094 (3)	
H19A	−0.035336	1.217659	−0.703384	0.112*	
H19B	−0.164525	1.187025	−0.657277	0.112*	
C20	0.4050 (6)	0.8819 (5)	−0.4499 (3)	0.0451 (14)	
C21	0.4509 (7)	0.9660 (5)	−0.3654 (4)	0.0616 (18)	
H21A	0.415495	0.928486	−0.318813	0.092*	
H21B	0.412063	1.049886	−0.372845	0.092*	
H21C	0.554710	0.973733	−0.350127	0.092*	
C22	0.6615 (6)	0.6425 (5)	−0.4229 (4)	0.0444 (14)	
C23	0.7439 (9)	0.5099 (7)	−0.5426 (5)	0.0636 (19)	
C24	0.8561 (9)	0.5668 (10)	−0.5836 (6)	0.086 (2)	
HN2	0.547 (5)	0.304 (4)	−0.126 (3)	0.026 (14)*	
HN3	0.620 (5)	0.374 (4)	0.080 (3)	0.025 (15)*	
HN5	0.571 (7)	0.752 (6)	−0.348 (5)	0.08 (2)*	
HN6	0.599 (7)	0.655 (6)	−0.542 (4)	0.07 (2)*	
H2	0.367 (7)	0.206 (6)	0.097 (4)	0.08 (2)*	
H3	0.223 (7)	0.104 (6)	0.182 (5)	0.08 (2)*	
H6	0.243 (7)	−0.078 (6)	−0.097 (4)	0.07 (2)*	
H11A	0.694 (5)	0.549 (4)	0.167 (3)	0.028 (13)*	
H11B	0.757 (6)	0.600 (6)	0.094 (4)	0.064 (19)*	
H12A	0.946 (6)	0.449 (5)	0.121 (4)	0.051 (18)*	
H12B	0.968 (8)	0.563 (7)	0.197 (5)	0.12 (3)*	
H12C	0.886 (7)	0.400 (6)	0.201 (4)	0.08 (2)*	
H14	0.252 (7)	1.096 (6)	−0.469 (4)	0.07 (2)*	
H17	0.043 (7)	0.792 (6)	−0.702 (4)	0.08 (2)*	
H18	0.260 (6)	0.752 (6)	−0.589 (4)	0.060 (19)*	
H23A	0.774 (6)	0.460 (6)	−0.493 (4)	0.065 (19)*	
H23B	0.683 (7)	0.460 (6)	−0.584 (5)	0.09 (2)*	
H24A	0.792 (7)	0.631 (6)	−0.640 (5)	0.09 (2)*	
H24B	0.924 (8)	0.624 (6)	−0.537 (5)	0.09 (2)*	
H24C	0.931 (9)	0.500 (8)	−0.616 (6)	0.13 (3)*	
Cl1	0.42642 (16)	0.68707 (14)	−0.23721 (9)	0.0516 (4)	
Cl2	0.4929 (2)	0.31509 (14)	−0.29256 (10)	0.0685 (5)	
Hg1	0.62812 (3)	0.52412 (2)	−0.23243 (2)	0.05213 (10)	
N1	0.4794 (5)	0.2433 (4)	−0.0241 (3)	0.0445 (11)	
N2	0.5602 (5)	0.3131 (4)	−0.0711 (3)	0.0429 (12)	
N3	0.6593 (5)	0.4255 (5)	0.0609 (3)	0.0427 (12)	
N4	0.4667 (5)	0.7771 (5)	−0.4701 (3)	0.0472 (12)	
N5	0.5754 (5)	0.7378 (5)	−0.4026 (3)	0.0503 (13)	
N6	0.6554 (6)	0.6068 (5)	−0.5071 (3)	0.0524 (13)	
O1	0.0695 (6)	−0.1239 (6)	0.1266 (4)	0.0980 (17)	
O2	0.0884 (6)	−0.2141 (4)	−0.0112 (4)	0.0935 (17)	
O3	0.0275 (5)	1.1772 (4)	−0.5793 (3)	0.0834 (15)	
O4	−0.0707 (6)	1.0307 (5)	−0.6972 (4)	0.0996 (18)	
S1	0.75042 (16)	0.50784 (14)	−0.07567 (9)	0.0506 (4)	
S2	0.78087 (16)	0.58069 (17)	−0.33820 (10)	0.0586 (4)	

### Bis{(E)-2-[1-(benzo[d][1,3]dioxol-5-yl)ethylidene]-N-ethylhydrazine-1-carbothioamide-κS}dichloridomercury(II) Atomic displacement parameters (Å^2^)

**Table:**

	*U*^11^	*U*^22^	*U*^33^	*U*^12^	*U*^13^	*U*^23^
C1	0.049 (4)	0.044 (3)	0.054 (4)	−0.002 (3)	0.011 (3)	0.008 (3)
C2	0.092 (6)	0.077 (5)	0.055 (5)	−0.032 (4)	0.015 (4)	0.001 (4)
C3	0.107 (7)	0.117 (7)	0.062 (5)	−0.051 (6)	0.026 (5)	0.001 (5)
C4	0.065 (5)	0.074 (5)	0.074 (5)	−0.007 (4)	0.015 (4)	0.036 (4)
C5	0.047 (4)	0.042 (3)	0.088 (5)	−0.004 (3)	0.019 (4)	0.014 (3)
C6	0.054 (4)	0.052 (4)	0.067 (5)	−0.002 (3)	0.017 (4)	0.001 (4)
C7	0.078 (6)	0.058 (5)	0.185 (10)	0.007 (4)	0.053 (6)	0.032 (6)
C8	0.050 (4)	0.044 (3)	0.043 (3)	0.005 (3)	0.006 (3)	0.006 (3)
C9	0.128 (7)	0.079 (5)	0.050 (4)	−0.043 (5)	0.022 (4)	−0.007 (4)
C10	0.036 (3)	0.042 (3)	0.036 (3)	0.006 (2)	0.004 (2)	0.012 (2)
C11	0.055 (4)	0.056 (4)	0.036 (3)	−0.002 (3)	0.009 (3)	0.001 (3)
C12	0.055 (5)	0.082 (5)	0.065 (5)	0.003 (4)	0.012 (4)	0.010 (4)
C13	0.051 (4)	0.051 (4)	0.045 (4)	−0.003 (3)	0.010 (3)	0.009 (3)
C14	0.055 (4)	0.044 (4)	0.055 (4)	−0.004 (3)	0.013 (3)	0.008 (3)
C15	0.051 (4)	0.055 (4)	0.065 (4)	0.005 (3)	0.012 (3)	0.015 (3)
C16	0.064 (5)	0.073 (5)	0.064 (5)	0.007 (4)	0.000 (4)	0.010 (4)
C17	0.092 (6)	0.079 (5)	0.073 (5)	0.007 (5)	−0.019 (5)	−0.006 (4)
C18	0.079 (5)	0.063 (5)	0.063 (5)	0.015 (4)	−0.005 (4)	0.002 (4)
C19	0.070 (5)	0.074 (5)	0.131 (8)	0.009 (4)	−0.003 (5)	0.031 (5)
C20	0.057 (4)	0.047 (3)	0.034 (3)	−0.008 (3)	0.013 (3)	0.010 (3)
C21	0.079 (5)	0.051 (4)	0.050 (4)	0.004 (3)	0.003 (3)	0.000 (3)
C22	0.042 (3)	0.050 (3)	0.044 (4)	−0.001 (3)	0.013 (3)	0.011 (3)
C23	0.075 (5)	0.069 (5)	0.046 (4)	0.010 (4)	0.013 (4)	−0.002 (4)
C24	0.067 (5)	0.118 (7)	0.083 (6)	0.016 (5)	0.037 (5)	−0.003 (6)
Cl1	0.0573 (9)	0.0581 (9)	0.0434 (8)	0.0080 (7)	0.0164 (7)	0.0111 (7)
Cl2	0.1078 (14)	0.0508 (9)	0.0429 (9)	−0.0072 (9)	0.0067 (9)	0.0045 (7)
Hg1	0.06164 (17)	0.05620 (15)	0.04099 (14)	0.00356 (10)	0.01264 (10)	0.01320 (10)
N1	0.049 (3)	0.046 (3)	0.038 (3)	−0.001 (2)	0.006 (2)	0.011 (2)
N2	0.048 (3)	0.048 (3)	0.031 (3)	−0.001 (2)	0.005 (2)	0.005 (2)
N3	0.043 (3)	0.050 (3)	0.036 (3)	−0.010 (2)	0.007 (2)	0.010 (2)
N4	0.052 (3)	0.055 (3)	0.036 (3)	0.009 (2)	0.009 (2)	0.011 (2)
N5	0.056 (3)	0.063 (3)	0.032 (3)	0.005 (3)	0.008 (3)	0.011 (3)
N6	0.061 (3)	0.057 (3)	0.041 (3)	0.011 (3)	0.013 (3)	0.006 (3)
O1	0.094 (4)	0.109 (4)	0.100 (4)	−0.033 (3)	0.031 (3)	0.038 (3)
O2	0.088 (4)	0.057 (3)	0.146 (5)	−0.017 (3)	0.051 (4)	0.007 (3)
O3	0.077 (3)	0.070 (3)	0.099 (4)	0.023 (3)	0.000 (3)	0.022 (3)
O4	0.079 (4)	0.099 (4)	0.106 (4)	0.021 (3)	−0.021 (3)	0.017 (3)
S1	0.0488 (9)	0.0633 (9)	0.0398 (8)	−0.0082 (7)	0.0055 (7)	0.0165 (7)
S2	0.0478 (9)	0.0863 (12)	0.0470 (9)	0.0118 (8)	0.0132 (7)	0.0258 (8)

### Bis{(E)-2-[1-(benzo[d][1,3]dioxol-5-yl)ethylidene]-N-ethylhydrazine-1-carbothioamide-κS}dichloridomercury(II) Geometric parameters (Å, º)

**Table:**

C1—C2	1.392 (9)	C15—C16	1.357 (9)
C1—C6	1.403 (8)	C15—O3	1.381 (7)
C1—C8	1.483 (8)	C16—C17	1.359 (10)
C2—C3	1.406 (10)	C16—O4	1.391 (8)
C2—H2	0.99 (6)	C17—C18	1.404 (9)
C3—C4	1.358 (10)	C17—H17	1.05 (6)
C3—H3	0.95 (7)	C18—H18	0.91 (6)
C4—O1	1.366 (8)	C19—O3	1.415 (8)
C4—C5	1.368 (9)	C19—O4	1.429 (8)
C5—C6	1.340 (9)	C19—H19A	0.9700
C5—O2	1.383 (7)	C19—H19B	0.9700
C6—H6	0.87 (6)	C20—N4	1.293 (7)
C7—O1	1.410 (10)	C20—C21	1.495 (7)
C7—O2	1.424 (10)	C21—H21A	0.9600
C7—H7A	0.9700	C21—H21B	0.9600
C7—H7B	0.9700	C21—H21C	0.9600
C8—N1	1.281 (6)	C22—N6	1.315 (7)
C8—C9	1.493 (8)	C22—N5	1.353 (7)
C9—H9A	0.9600	C22—S2	1.720 (6)
C9—H9B	0.9600	C23—N6	1.466 (8)
C9—H9C	0.9600	C23—C24	1.478 (11)
C10—N3	1.312 (7)	C23—H23A	0.96 (6)
C10—N2	1.349 (7)	C23—H23B	0.90 (7)
C10—S1	1.725 (5)	C24—H24A	1.21 (7)
C11—N3	1.464 (7)	C24—H24B	1.01 (7)
C11—C12	1.504 (9)	C24—H24C	1.16 (9)
C11—H11A	0.97 (5)	Cl1—Hg1	2.5681 (15)
C11—H11B	0.95 (6)	Cl2—Hg1	2.5133 (15)
C12—H12A	0.92 (6)	Hg1—S1	2.4832 (14)
C12—H12B	1.16 (8)	Hg1—S2	2.4878 (16)
C12—H12C	1.01 (7)	N1—N2	1.390 (6)
C13—C18	1.381 (8)	N2—HN2	0.83 (5)
C13—C14	1.393 (8)	N3—HN3	0.76 (5)
C13—C20	1.485 (8)	N4—N5	1.399 (6)
C14—C15	1.357 (8)	N5—HN5	0.85 (7)
C14—H14	0.84 (6)	N6—HN6	0.87 (6)
			
C2—C1—C6	118.3 (6)	C16—C17—H17	118 (3)
C2—C1—C8	120.8 (5)	C18—C17—H17	124 (3)
C6—C1—C8	121.0 (6)	C13—C18—C17	121.9 (7)
C1—C2—C3	122.3 (7)	C13—C18—H18	117 (4)
C1—C2—H2	116 (4)	C17—C18—H18	121 (4)
C3—C2—H2	121 (4)	O3—C19—O4	108.4 (5)
C4—C3—C2	116.9 (8)	O3—C19—H19A	110.0
C4—C3—H3	124 (4)	O4—C19—H19A	110.0
C2—C3—H3	119 (4)	O3—C19—H19B	110.0
C3—C4—O1	127.4 (8)	O4—C19—H19B	110.0
C3—C4—C5	120.7 (7)	H19A—C19—H19B	108.4
O1—C4—C5	111.9 (6)	N4—C20—C13	116.1 (5)
C6—C5—C4	123.8 (6)	N4—C20—C21	124.8 (5)
C6—C5—O2	128.3 (7)	C13—C20—C21	119.1 (5)
C4—C5—O2	107.9 (6)	C20—C21—H21A	109.5
C5—C6—C1	118.0 (7)	C20—C21—H21B	109.5
C5—C6—H6	118 (4)	H21A—C21—H21B	109.5
C1—C6—H6	123 (4)	C20—C21—H21C	109.5
O1—C7—O2	108.6 (6)	H21A—C21—H21C	109.5
O1—C7—H7A	110.0	H21B—C21—H21C	109.5
O2—C7—H7A	110.0	N6—C22—N5	118.0 (5)
O1—C7—H7B	110.0	N6—C22—S2	123.2 (5)
O2—C7—H7B	110.0	N5—C22—S2	118.7 (4)
H7A—C7—H7B	108.4	N6—C23—C24	113.1 (6)
N1—C8—C1	115.6 (5)	N6—C23—H23A	102 (4)
N1—C8—C9	125.4 (5)	C24—C23—H23A	118 (4)
C1—C8—C9	119.0 (5)	N6—C23—H23B	106 (5)
C8—C9—H9A	109.5	C24—C23—H23B	109 (5)
C8—C9—H9B	109.5	H23A—C23—H23B	107 (6)
H9A—C9—H9B	109.5	C23—C24—H24A	106 (3)
C8—C9—H9C	109.5	C23—C24—H24B	109 (4)
H9A—C9—H9C	109.5	H24A—C24—H24B	110 (5)
H9B—C9—H9C	109.5	C23—C24—H24C	120 (4)
N3—C10—N2	118.1 (5)	H24A—C24—H24C	108 (5)
N3—C10—S1	119.7 (4)	H24B—C24—H24C	105 (6)
N2—C10—S1	122.3 (4)	S1—Hg1—S2	117.71 (5)
N3—C11—C12	112.5 (6)	S1—Hg1—Cl2	109.19 (5)
N3—C11—H11A	106 (3)	S2—Hg1—Cl2	107.93 (6)
C12—C11—H11A	112 (3)	S1—Hg1—Cl1	109.03 (5)
N3—C11—H11B	110 (3)	S2—Hg1—Cl1	108.61 (5)
C12—C11—H11B	113 (4)	Cl2—Hg1—Cl1	103.42 (6)
H11A—C11—H11B	102 (4)	C8—N1—N2	118.3 (5)
C11—C12—H12A	111 (4)	C10—N2—N1	117.2 (5)
C11—C12—H12B	107 (4)	C10—N2—HN2	120 (3)
H12A—C12—H12B	97 (5)	N1—N2—HN2	122 (3)
C11—C12—H12C	112 (4)	C10—N3—C11	126.7 (5)
H12A—C12—H12C	109 (5)	C10—N3—HN3	115 (4)
H12B—C12—H12C	118 (5)	C11—N3—HN3	118 (4)
C18—C13—C14	119.0 (6)	C20—N4—N5	115.1 (5)
C18—C13—C20	119.3 (6)	C22—N5—N4	118.5 (5)
C14—C13—C20	121.7 (5)	C22—N5—HN5	117 (5)
C15—C14—C13	118.3 (6)	N4—N5—HN5	121 (5)
C15—C14—H14	119 (4)	C22—N6—C23	126.3 (6)
C13—C14—H14	123 (4)	C22—N6—HN6	112 (4)
C16—C15—C14	122.2 (6)	C23—N6—HN6	121 (4)
C16—C15—O3	110.2 (6)	C4—O1—C7	105.2 (6)
C14—C15—O3	127.5 (6)	C5—O2—C7	106.4 (6)
C15—C16—C17	121.9 (6)	C15—O3—C19	105.1 (5)
C15—C16—O4	109.8 (6)	C16—O4—C19	104.5 (5)
C17—C16—O4	128.3 (6)	C10—S1—Hg1	110.21 (18)
C16—C17—C18	116.7 (7)	C22—S2—Hg1	104.2 (2)
			
C6—C1—C2—C3	0.4 (11)	C14—C13—C20—C21	10.0 (8)
C8—C1—C2—C3	179.7 (7)	C1—C8—N1—N2	−178.1 (4)
C1—C2—C3—C4	−0.9 (13)	C9—C8—N1—N2	2.4 (9)
C2—C3—C4—O1	179.8 (7)	N3—C10—N2—N1	3.7 (7)
C2—C3—C4—C5	1.3 (13)	S1—C10—N2—N1	−177.4 (4)
C3—C4—C5—C6	−1.3 (12)	C8—N1—N2—C10	−171.7 (5)
O1—C4—C5—C6	179.9 (6)	N2—C10—N3—C11	179.6 (5)
C3—C4—C5—O2	178.7 (7)	S1—C10—N3—C11	0.7 (8)
O1—C4—C5—O2	0.0 (8)	C12—C11—N3—C10	−93.9 (8)
C4—C5—C6—C1	0.8 (10)	C13—C20—N4—N5	−174.3 (5)
O2—C5—C6—C1	−179.2 (6)	C21—C20—N4—N5	5.8 (8)
C2—C1—C6—C5	−0.4 (9)	N6—C22—N5—N4	11.4 (8)
C8—C1—C6—C5	−179.7 (6)	S2—C22—N5—N4	−172.4 (4)
C2—C1—C8—N1	5.8 (9)	C20—N4—N5—C22	−168.8 (5)
C6—C1—C8—N1	−174.9 (5)	N5—C22—N6—C23	177.1 (6)
C2—C1—C8—C9	−174.6 (6)	S2—C22—N6—C23	1.1 (9)
C6—C1—C8—C9	4.7 (9)	C24—C23—N6—C22	−106.9 (8)
C18—C13—C14—C15	−1.3 (9)	C3—C4—O1—C7	179.6 (8)
C20—C13—C14—C15	179.8 (6)	C5—C4—O1—C7	−1.7 (8)
C13—C14—C15—C16	1.5 (10)	O2—C7—O1—C4	2.8 (8)
C13—C14—C15—O3	179.6 (6)	C6—C5—O2—C7	−178.1 (7)
C14—C15—C16—C17	−0.8 (12)	C4—C5—O2—C7	1.8 (8)
O3—C15—C16—C17	−179.2 (7)	O1—C7—O2—C5	−2.9 (8)
C14—C15—C16—O4	178.1 (6)	C16—C15—O3—C19	−8.3 (8)
O3—C15—C16—O4	−0.3 (8)	C14—C15—O3—C19	173.4 (7)
C15—C16—C17—C18	0.0 (13)	O4—C19—O3—C15	13.6 (8)
O4—C16—C17—C18	−178.8 (7)	C15—C16—O4—C19	8.6 (8)
C14—C13—C18—C17	0.5 (11)	C17—C16—O4—C19	−172.5 (9)
C20—C13—C18—C17	179.5 (7)	O3—C19—O4—C16	−13.7 (8)
C16—C17—C18—C13	0.2 (13)	N3—C10—S1—Hg1	−153.3 (4)
C18—C13—C20—N4	11.1 (8)	N2—C10—S1—Hg1	27.9 (5)
C14—C13—C20—N4	−170.0 (5)	N6—C22—S2—Hg1	−132.1 (5)
C18—C13—C20—C21	−168.9 (6)	N5—C22—S2—Hg1	52.0 (5)

### Bis{(E)-2-[1-(benzo[d][1,3]dioxol-5-yl)ethylidene]-N-ethylhydrazine-1-carbothioamide-κS}dichloridomercury(II) Hydrogen-bond geometry (Å, º)

**Table:**

*D*—H···*A*	*D*—H	H···*A*	*D*···*A*	*D*—H···*A*
C7—H7*B*···S1^i^	0.97	2.85	3.642 (8)	139
C9—H9*C*···Cl2	0.96	2.89	3.474 (7)	121
C21—H21*A*···Cl1	0.96	2.90	3.675 (6)	139
C21—H21*B*···Cl2^ii^	0.96	2.95	3.707 (6)	136
N2—H*N*2···Cl2	0.83 (5)	2.53 (5)	3.354 (5)	170 (4)
N3—H*N*3···Cl1^iii^	0.76 (5)	2.68 (5)	3.293 (5)	140 (4)
N3—H*N*3···N1	0.76 (5)	2.22 (5)	2.604 (6)	113 (4)
N5—H*N*5···Cl1	0.85 (7)	2.53 (7)	3.233 (6)	141 (6)
N6—H*N*6···Cl2^iv^	0.87 (6)	2.58 (6)	3.300 (5)	141 (5)
N6—H*N*6···N4	0.87 (6)	2.19 (6)	2.649 (7)	112 (5)
C23—H23*A*···S2	0.96 (6)	2.60 (6)	3.134 (8)	115 (4)
C24—H24*A*···Cl2^iv^	1.21 (7)	2.77 (7)	3.739 (9)	137 (4)
C24—H24*B*···O3^v^	1.01 (7)	2.60 (7)	3.513 (11)	151 (6)

Symmetry codes: (i) −*x*+1, −*y*, −*z*; (ii) *x*, *y*+1, *z*; (iii) −*x*+1, −*y*+1, −*z*; (iv) −*x*+1, −*y*+1, −*z*−1; (v) −*x*+1, −*y*+2, −*z*−1.
