# Supplementary material for: Synthesis, crystal structure and Hirshfeld analysis of the bis­{(E)-2-[1-(benzo[d][1,3]dioxol-5-yl)ethylidene]-N-ethyl­hydrazine-1-carbo­thio­amide-κS}di­chlorido­mercury(II) complex
Source: Acta Crystallogr E Crystallogr Commun. 2026 May 15;82(Pt 6):656–64. doi: 10.1107/S2056989026004822 (PMC13238988; doi:10.1107/S2056989026004822)
Supplement: Supplementary file 3 [file e-82-00656-sup3.pdf]

SUPPORTING INFORMATION

Synthesis, crystal structure and Hirshfeld analysis of the dichloridobis{(*E*)-2-[1-(benzo[*d*][1,3]dioxol-5-yl)ethylidene]-*N*-ethylhydrazine-1-carbothioamide-κ*S*}mercury(II) complex

Renan Lira de Farias,[a] Johannes Beck,[b] Jörg Daniels[b] and Adriano Bof de Oliveira[c*]

[a]Departamento de Química, Pontifícia Universidade Católica do Rio de Janeiro, Rua Marquês de São Vicente 225, 22451-900 Rio de Janeiro-RJ, Brazil

[b]Institut für Anorganische Chemie, Rheinische Friedrich-Wilhelms-Universität Bonn, Gerhard-Domagk-Strasse 1, D-53121 Bonn, Germany

[c]Núcleo de Química, Universidade Federal do Rio Grande, Avenida Itália km 08, 96203-900 Rio Grande-RS, Brazil

Correspondence e-mail: adriano@furg.br

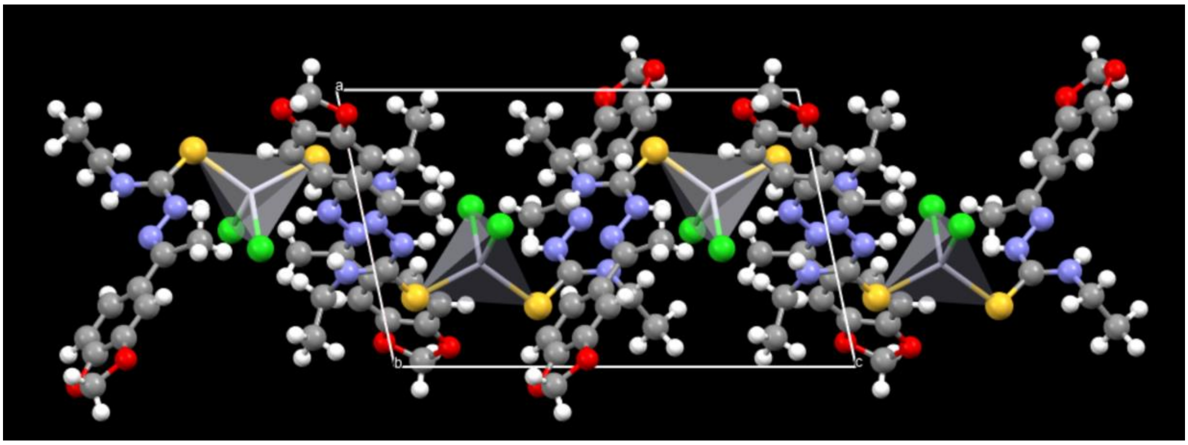

Figure SI 1: Crystal section of **$HgCl_2(TSC1)_2$** viewed from the *b*-axis (this work). The supramolecular arrangement resembles a *zigzag* pattern along the *c*-axis. The coordination polyhedra are drawn with 70% transparency and the figure is simplified for clarity.

[**TSC1** is 3',4'-(methylenedioxy)acetophenone 4-ethylthiosemicarbazone; CUCZUX;

Oliveira *et al.* (2015)]

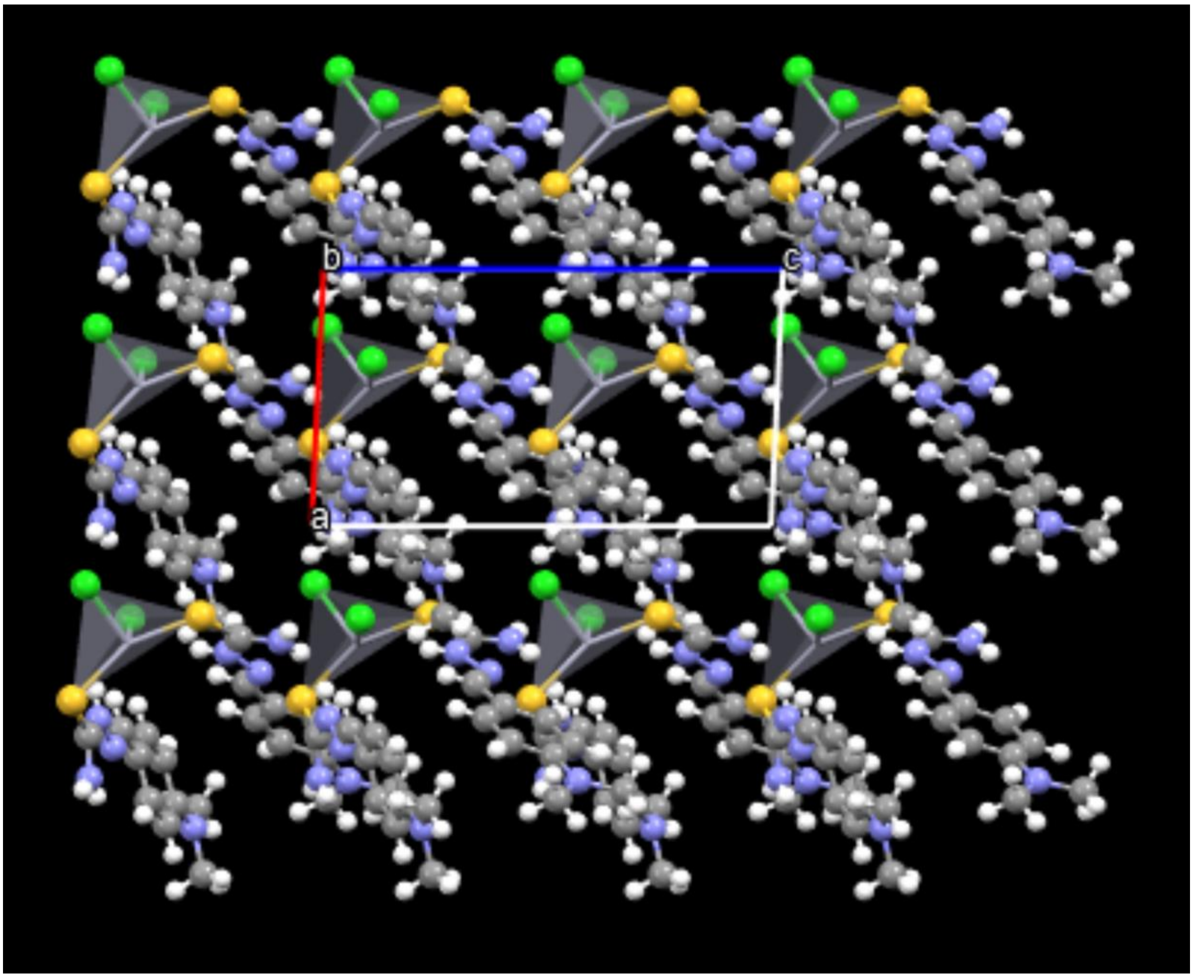

Figure SI 2: Crystal section of $\mathbf{HgCl_2(TSC2)_2}$ viewed from the *b*-axis (EFUKEX; Trzesowska-Kruszynska, 2014). The supramolecular arrangement resembles a tape-like structure along the *ac*-plane. The coordination polyhedra are drawn with 70% transparency and the figure is simplified for clarity.

[**TSC2** is *p*-dimethylaminobenzaldehyde thiosemicarbazone]

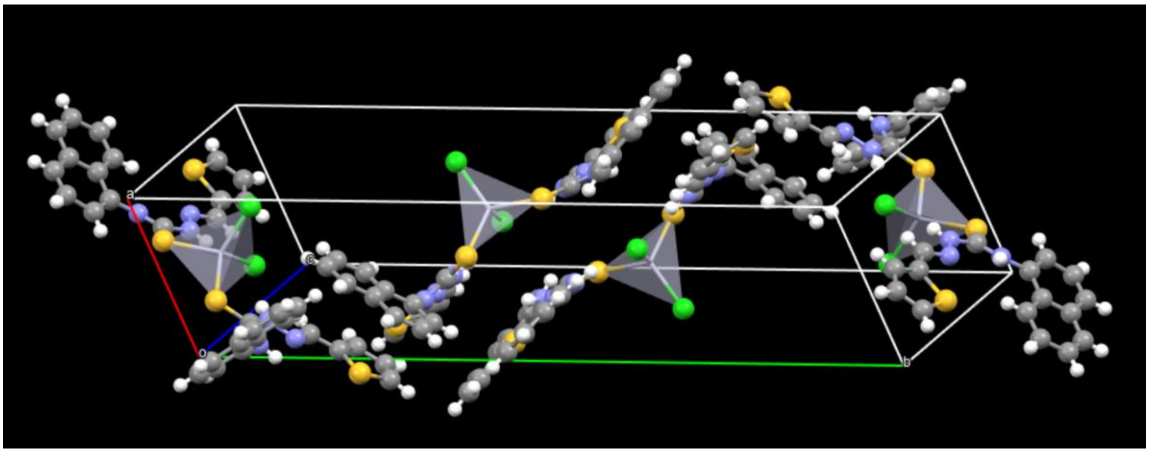

Figure SI 3: Crystal section of **$HgCl_2(TSC3)_2$** (IRETOP; Basu & Das, 2011). Neither strong nor relevant intermolecular interactions are observed between the molecules and only weak intermolecular interactions, *e.g.*, the London dispersion forces can be suggested. The coordination polyhedra are drawn with 70% transparency and the figure is simplified for clarity.

[**TSC3** is 2-thiophenealdehyde-*N*-(4)-napthylthiosemicarbazone]

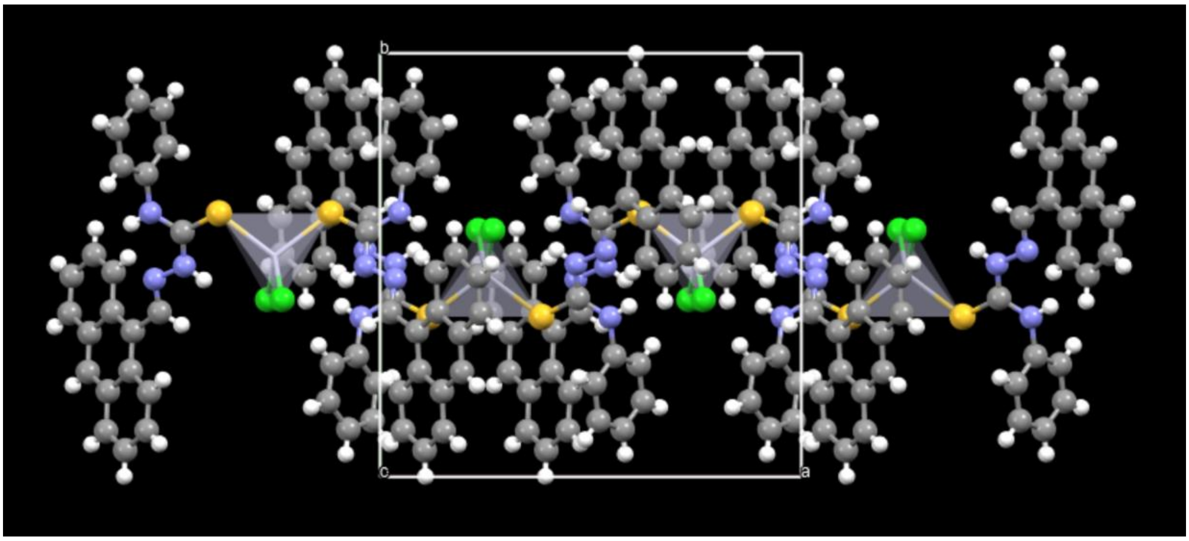

Figure SI 4: Crystal section of **$HgCl_2(TSC4)_2$** viewed from the *c*-axis (MOCXAH; Nath & Baruah, 2023). The supramolecular arrangement resembles a *zigzag* pattern along the *a*-axis. The coordination polyhedra are drawn with 70% transparency and the figure is simplified for clarity.

[**TSC4** is 2-(anthracen-9-ylmethylene)-*N*-phenylthiosemicarbazone]
